# Electrical Stimulation of Cells: Drivers, Technology, and Effects

**DOI:** 10.1021/acs.chemrev.4c00468

**Published:** 2025-07-17

**Authors:** Kian Kadan-Jamal, Filip Wronowski, Amy Jin, Tobias E. Naegele, Viviana Rincón Montes, Xudong Tao, Antonio Dominguez-Alfaro, Chaeyeon Lee, George G. Malliaras

**Affiliations:** † Electrical Engineering, Division Department of Engineering, 2152 University of Cambridge, Cambridge CB3 0FA, U.K.; ‡ Institute of Biological Information Processing (IBI-3) − Bioelectronics, Forschungszentrum, Leo-Brandt-Str., D-52425 Jülich, Germany; § Institute of Microelectronics of Seville, Spanish National Research Council (CSIC), 41092 Sevilla, Spain

## Abstract

Exposure of cells to electric fields is used in applications as diverse as deep brain stimulation to treat the symptoms of Parkinson’s disease, tumor-treating fields to delay the progression of hard-to-treat cancers, and electroporation to deliver genetic materials to cells. It is also used to study the fundamental properties of electrically active cells and to induce changes in cell behavior, such as the directed outgrowth of neurites, that may one day find applications in the clinic. This review discusses some of the effects elicited on cells upon exposure to electric fields, both acutely and at longer time scales, and considers the underlying mechanisms proposed. It also provides an overview of the technology used to study the effects of exposure of cell to electric fields, including the different types of metal/electrolyte interfaces and the electrode materials used in *in vitro* and *in vivo* applications. The aim is to bring together concepts from different communities to highlight similarities, identify potential synergies, and create common ground that may lead to cross-fertilization and advances in the field.

## Graphical abstract

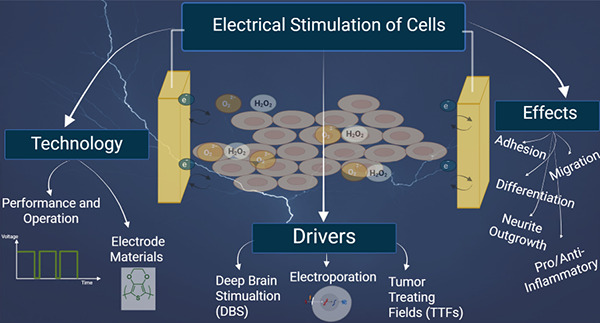

## 1 Introduction

The use of electricity to elicit health benefits goes back to antiquity,^1–3^ when electric fish were employed to treat migraines.^4^ Today, electrical stimulation is used in bioelectronic medicine to treat conditions as diverse as heart arrhythmias, hearing and vision loss, chronic pain, spinal cord injury, movement disorders, epilepsy, depression, and rheumatoid arthritis.^1^ Various communities deal with understanding and using the phenomena elicited by electrical stimulation of cells, and different names are used to describe these communities or the phenomena they investigate.^5,6^ The neuromodulation community, for example, is interested in the excitatory or inhibitory effects of electrical stimulation on the nervous system.^7–10^ These effects are elicited by changes in the polarization of the neuronal cell membranes.^11^ Another community deals with Tumor Treating Fields (TTF),^12–14^ which are applied to the body to delay the progression of some hard-to-treat cancers. Molecular polarization effects are believed to play a key role in this domain. The electroporation community uses high voltage pulses to temporarily permeabilize cell membranes, enabling the delivery of genetic material and other molecules into cells.^15,16^ Electrical stimulation is also used to study fundamental properties of electrically active cells and to elicit changes in cell behavior including cell adhesion, migration, differentiation, neurite outgrowth, and pro- or anti-inflammatory effects. Mechanisms under investigation include electrophoresis of proteins, effects on calcium signal pathways, and transcriptional changes. It is understood that non-neuronal cells also play a role in some of these phenomena. Finally, some of these effects are elicited with particular electrodes, which highlights the issues associated with applying well-defined electric field in electrolytes.

The technology used for electrical stimulation of cells varies widely within the research community.^17^ Different configurations and materials are used, which makes comparisons of results obtained by different laboratories difficult.^18^ Electrodes that operate in the capacitive regime avoid electrochemical reactions that produce species that may interfere with the cells. However, these electrodes are not suitable for low frequency or DC (direct current) stimulation, limiting their application to the study of high frequency phenomena. To access the low frequency regime, electrodes operating in the Faradaic regime are used, and some strategies have been developed to mitigate the effects of electrochemical reactions at electrode/electrolyte interfaces. Different metrics are used to characterize electrode performance, including impedance, and charge injection and storage capacity, while the stability of electrodes and their interaction with tissues is attracting a great deal of attention.

### 1.1 Drivers and Application Examples

Neurological disorders affect over 1.3 billion people worldwide, imposing significant global health, economic and societal burden.^19^ Multiple conditions including stroke, cancer, neurodegeneration, and traumatic injury contribute to this burden. Bioelectronic medicine is emerging as a cutting-edge therapeutic approach,^20–23^ utilizing implantable^24,25^ and cutaneous^26–29^ electronic devices to restore or replace neurological functions. These devices have already transformed patient care across various medical fields. Examples of neurotechnology applications include vagus nerve stimulators for epilepsy,^30,31^ deep brain stimulators for Parkinson’s disease,^32^ auditory brainstem or cochlear implants for hearing disorders,^33^ and spinal cord stimulators for refractory neuropathic pain.^34^ Emerging devices that include brain-computer interfaces for restoring mobility^35^ and “electroceuticals” that treat an expanding array of conditions,^21^ paint a bright future of bioelectronic medicine. Importantly, the physical phenomena that occur upon stimulation are being investigated.^36–39^ Finally, noncontact stimulation techniques such as Transcranial Magnetic Stimulation (TMS),^40^ offer an alternative approach by employing electromagnetic induction to modulate neural activity in the brain.

We set the stage for this review by briefly discussing three applications of electrical stimulation technologies: Deep Brain stimulation (DBS), electroporation, and Tumor Treating Fields (TTFs). These examples were chosen to present a spectrum of technologies, from well-established clinical techniques backed by extensive scientific evidence (DBS and electroporation) to newer, emerging approaches (TTF). While TTF currently has smaller evidence base compared to DBS and electroporation, it offers unique mechanisms and therapeutic potential making it a valuable addition to this discussion.

#### 1.1.1 Deep Brain Stimulation (DBS)

Deep Brain Stimulation (DBS),^25,41–48^ is one example of an application of bioelectronic medicine that the United States Food and Drug Administration (FDA) has approved for the treatment of Parkinson’s disease. DBS has largely replaced ablative neurosurgery for patients with movement disorders,^49^ such as essential tremor and dystonia.^50^ It has also demonstrated efficacy against treatment-resistant depression,^51^ and obsessive-compulsive disorder (OCD).^52^ DBS involves the precise delivery of low frequency electrical impulses to specific regions of the brain, achieved through the use of surgically implanted electrodes. The process employs stereotactic techniques and neuroimaging to create small holes in the skull and precisely place the electrodes, which are connected to a subcutaneously implanted pulse generator. The stimulation parameters can be adjusted externally by clinicians and are typically set at frequencies of 60–130 Hz, voltage of a few Volts or current of a few mA, and pulse widths of 60–200 μs.^53^ For Parkinson’s disease, the therapeutic mechanism involves modulation and prevention of pathological activity through a complex pattern of facilitative and inhibitory effects,^45,46^ as stimulation may inhibit the cell body while activating the axons simultaneously.

Recent advancements in DBS technology, such as the use of segmented electrodes and field steering, have improved its specificity, leading to better treatment outcomes.^41^ The benefits of reversibility and adjustability of electrical simulation are particularly useful in this application.^54^ After surgical implantation, stimulation parameters such as the spatial location, intensity, and the temporal characteristics of the stimulation pattern can be tailored to individual requirements (e.g., modifications in medication).^45^ Despite these advancements, challenges remain in developing DBS devices that provide more targeted stimulation, limit the risk associated with the implantation,^55^ and require less frequent visits for battery replacement.^56^

#### 1.1.2 Electroporation

Electroporation transiently permeabilizes the cell membrane by exposing cells, either *in vitro* or *in vivo*, to high frequency electric field pulses.^15,57–59^ Stimulation may involve single, multiple, biphasic, or monophasic pulses that create temporary aqueous pores in the lipid bilayer, allowing the transport of therapeutic molecules such as nucleic acids, proteins, or chemotherapeutic agent into cells.^59,60^ Delivery of DNA has been demonstrated into a diverse array of mammalian cells,^61–63^ as well as in bacteria, yeasts, and plant cells. Beyond gene delivery, electroporation has found a significant therapeutic applications in as cancer treatment and vaccine development.^64^

Electroporation devices deliver high-voltage pulses to induce electric fields across the target tissue.^65^ The efficiency of electroporation depends on the field strength and the pulse length and shape.^57^ For mammalian cells, fields of 0.1 to 2 kV/cm^66^ are required. These values differ significantly for yeast and bacteria, where higher field strengths are often necessary due to differences in membrane properties and cell size.

Electroporation involves a combination of biophysical and electrochemical effects that disrupt the membrane’s structural integrity. The application of an electric field creates a voltage difference across the cell’s membrane. Once the transmembrane potential exceeds a critical threshold (typically 200–1000 mV), the bilayer destabilizes, resulting in the formation of transient or permanent aqueous pores.^67^ The dynamic nature of the pores allows them to reseal after the electric field is removed (reversible electroporation). Under higher fields the pores can remain open, leading to irreversible electroporation,^62,68^ with applications in nonthermal tissue ablation,^69^ particularly in pancreatic, liver and prostate cancers.^70^ Calcium electroporation has emerged as an innovative approach, include cell death selectively in cancer cells by overwhelming their calcium homeostasis mechanisms.^71,72^ An alternative to traditional electroporation is magneto-permeabilization, uses high-intensity pulsed magnetic fields to achieve transient membrane permeabilization.^73^

#### 1.1.3 Tumor Treating Fields (TTFs)

Tumor treating Fields (TTFs) are a promising cancer treatment modality.^12,74–76^ TTFs are delivered noninvasively through pairs of cutaneous electrodes affixed to the patient’s scalp or other locations based on the type of tumor to be treated.^14,77^ The electrodes deliver low-intensity electric fields in the intermediate frequency range (100–500 kHz), which are believed to interfere with organelle assembly and block cell division.^78^ TTFs have shown selective toxicity to cancer cells, making them a potential alternative to conventional treatments.^79^

This treatment approach has demonstrated potential in various types of tumors. Randomized phase 3 clinical trials have evaluated its efficacy in treating glioblastoma.^12,75^ The findings suggest that when combined with standard maintenance Temozolomide chemotherapy for patients with newly diagnosed GBM, it can improve both progression-free survival and overall survival rates.^80^ The American Society of Clinical Oncology (ASCO) and the Neuro-Oncology Society (NSO) have established new guidelines recommending the use of TTF therapy for patients with newly diagnosed supratentorial glioblastoma after undergoing chemoradiation.^81,82^ These guidelines emphasize the importance of standardized treatment planning and continuous administration to maximize the therapy’s efficacy. The electric field is applied to the brain using four transducer arrays, each comprised of nine insulated electrodes fixed to the patient’s shaved scalp and connected to a portable device that should be worn for 18 h every day.^12,83^

### 1.2 Historical Overview

Therapeutic uses of electricity extend across different cultures and time periods.^1,84–86^ Ancient civilizations used bracelets and necklaces made of materials such as amber, a material known to become electrostatically charged. Animals such as the electric eel and torpedo fish have been used for the treatment of migraines and gout.^87^ The work of Luigi Galvani in the latter part of the 18th century, showed that electrical stimulation causes muscle contractions.^88^ Key milestones in the 19th century included Bartholow’s first documented electrical stimulation of the human brain.^87^ The era of bioelectronic medicine started in the 20th century, with the first implantable pacemaker in the late 1950s for the treatment of cardiac arrhythmias.^89^ As one of the most widely recognized and utilized applications of electrical stimulation, cardiac pacemakers paved the way for subsequent advancement in the field. In the early 1960s, Liberson introduced the first clinical application of functional electrical stimulation (FES) utilizing an external device with surface electrodes and a heel switch to stimulate the peroneal nerve, enabling hemiplegic patients to overcome foot drop during the swing phase of gait.^90^ In the 1960s, Brindley and Lewin tested electrodes implanted on the visual cortex of a blind patient,^91^ marking the first clinical use of cortical implants for vision restoration. The introduction of cochlear implants in the late 1960s provided a new method to treat profound deafness, restoring hearing for many individuals.^92^ In the 1970s, spinal cord stimulation emerged as an effective treatment for chronic pain and other indications.^93,94^ The first successful demonstration of electroporation as a method for transferring DNA into cells was in the 1980s.^15^ During the 1980s, clinical devices using electrical stimulation to enhance wound healing were developed, proving effective against chronic, nonhealing wounds.^95,96^ In the 2000s, deep brain stimulation (DBS) became a revolutionary treatment for movement disorders such as Parkinson’s disease, tremor, and dystonia.^32^ In 2011, the FDA approved the tumor treating fields (TTFs) for the treatment of recurrent glioblastoma. In 2015, it was also approved as an adjuvant therapy for newly diagnosed glioblastoma,^13^ and in the 2020s, vagus nerve stimulation (VNS) was introduced as a treatment for rheumatoid arthritis, reducing inflammation and disease activity in patients.^24^ The future holds great promise as bioelectronic medicine expands toward new directions that include the treatment of neuropsychiatric disorders, cancer, and autoimmune diseases. Figure 1 provides a historical overview of key developments, from cellular-level interventions to complex therapeutic applications, selected to illustrate the breadth of the field.

**Figure 1:**
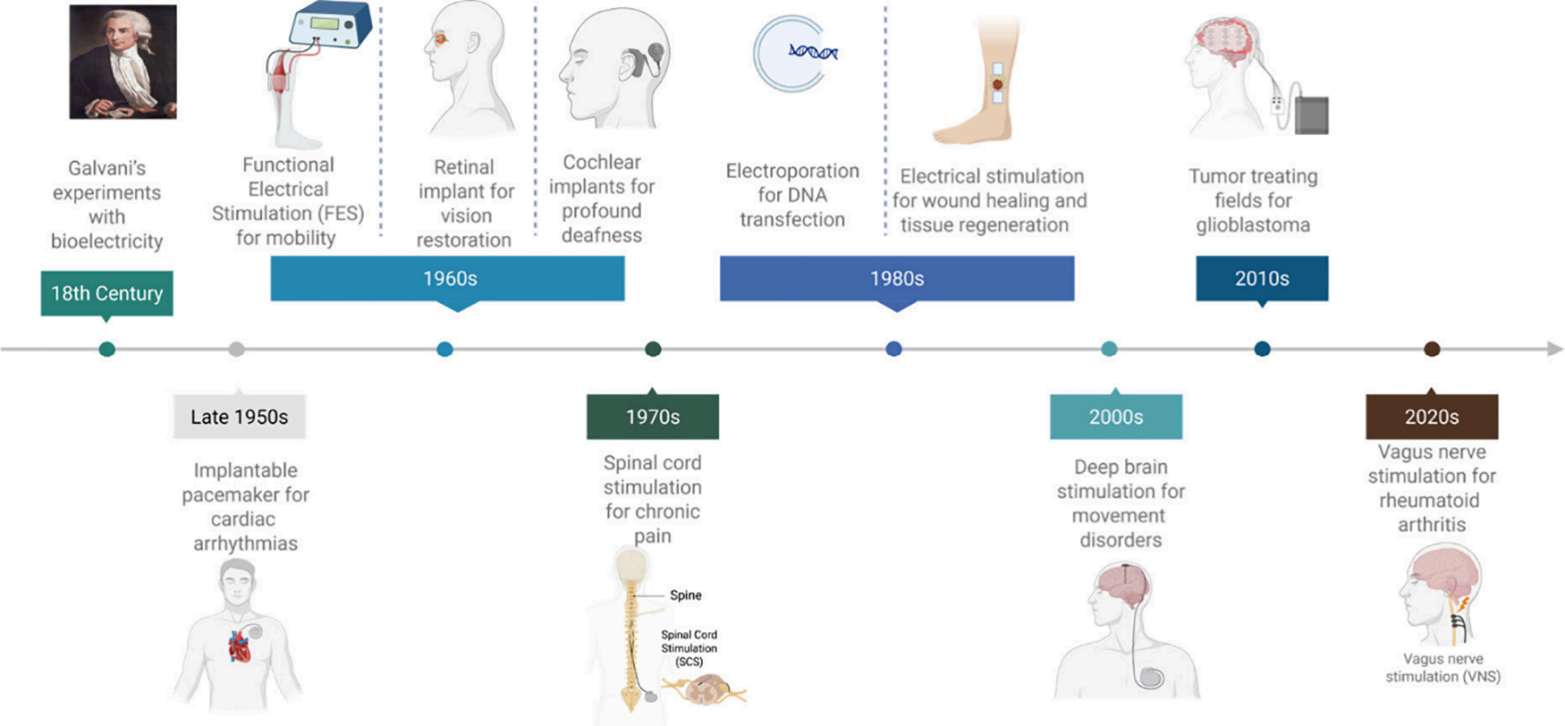
A historical overview of the uses of electrical stimulation in medicine, highlighting neuromodulation, cancer treatment and membrane manipulation to showcase diverse mechanisms and applications. Created in https://BioRender.com.

### 1.3 Scope of the Review

This review highlights the multitude of applications of electrical stimulation in biology, both *in vivo* and *in vitro*. In Section 2, we discuss electrode technologies, where the emphasis is on fundamental phenomena at metal/electrolyte interfaces as well as on the properties of the most commonly used electrode materials. In Section 3, we review the effects of exposure of cells to electric fields, with an emphasis on results obtained from *in vitro* experiments, as these represent “cleaner” experiments, without the complexities of the *in vivo* environment. Nevertheless, it is imperative to recognize that findings from *in vitro* studies may not always be directly applicable to *in vivo* conditions due to the differences in tissue complexity and the challenges associated with accurately replicating dosimetric conditions in a more physiologically relevant context. We focus on extracellular stimulation, and refer the reader to other papers for patch-micropipettes^97–101^ and nanoelectrodes.^102–104^ We also focus on single cell phenomena, as opposed to e.g. network activities or organism-level responses. Single cell phenomena are complex enough, and the mechanisms involved are many, hence limit the review to these to keep it tractable. We do not cover higher frequency effects, such as radio frequency tissue ablation.^105^ Finally, in Section 4, we summarize suggesting some directions for future work.

## 2 Technology for Electrical Stimulation

Unlike electronic devices that rely on the flow of electrons for their operation, cellular signaling involves the flow of ions.^106,107^ Electrodes are used to transduce between electronic and ionic currents and establish a bioelectronic communication channel. As a result, there are stringent requirements for the electrode materials used in terms of their ability to effectively achieve the transduction processes, their stability, and their biocompatibility. This section provides an overview of the electrode technologies used. We discuss electrode operating regimes, relevant performance metrics, and types of electrodes that are being used. The focus is on *in vitro* stimulation, which is used to understand the mechanisms of action, but the same principles apply to implantable configurations for e.g. DBS, and wearables ones for e.g. TTFs. The application of electric field stimulation in biological samples is illustrated in Figure 2, which presents different configuration for low-frequency, high frequency, and controlled DC simulation.

**Figure 2:**
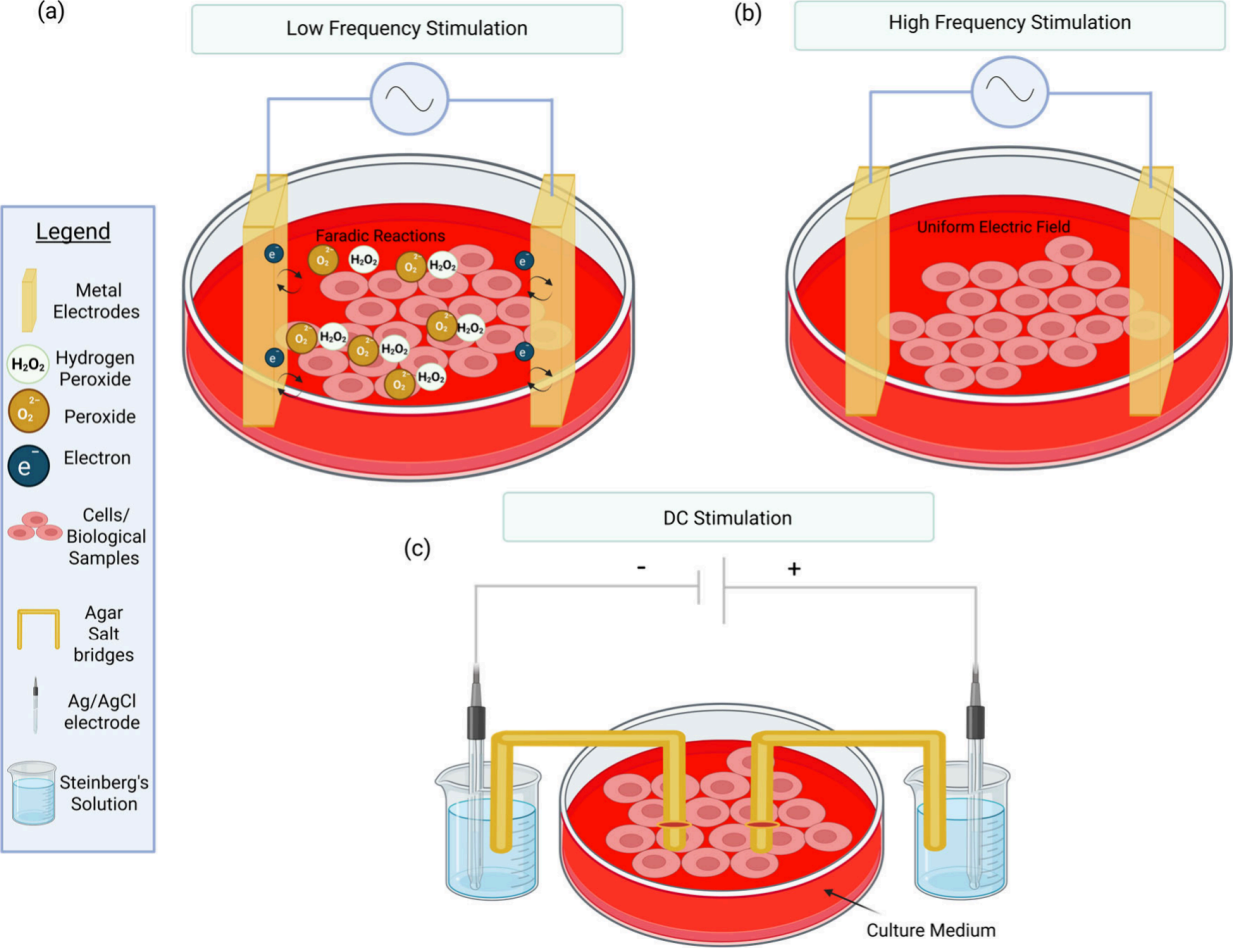
Schematic representation of setups used for in vitro electric field stimulation. (a) Low-frequency stimulation using metal electrodes, leading to Faradaic reactions, including the generation of hydrogen peroxide and other electrochemical byproducts. (b) High-frequency stimulation using metal electrodes. (c) DC stimulation with Ag/AgCl electrodes and salt bridges to minimize Faradaic reactions, enabling more controlled DC field application in the biological media. Created in https://BioRender.com.

### 2.1 Electrodes

To successfully stimulate cells, electronic to ionic transduction must occur at the electrode/electrolyte interface. Electrolyte in this context refers to a cell culture medium *in vitro*, the extracellular fluid in implantable applications, or a gel in a cutaneous application. Electrode operation can be described by two regimes, capacitive and Faradaic.^107^ These should be taken as idealized limits of electrode behavior, with real electrodes often exhibiting characteristics of both regimes.

#### 2.1.1 Capacitive Regime

In the capacitive regime, the application of a voltage leads to the accumulation of ions of the opposite charge at the electrode/electrolyte interface, i.e. a layer of anions at the positively biased electrode (anode) and a layer of cations at the cathode.^108^ In this reversible electrostatic process, electrons and ions are stored across the electrode surface but do not cross the electrode/electrolyte interface. Once ions accumulate near the interface, they effectively shield the applied potential, causing it to decay rapidly within a very short distance from the electrode. Only a limited number of charges can be stored at the electrode/electrolyte interface, determined by the applied voltage and the electrode capacitance. The latter depends on electrode’s nature, surface area and morphology. The spatial extent of the ion redistribution in the electrolyte can be roughly approximated by the Debye length. For example, in a 100 mM NaCl solution at room temperature, the Debye length is approximately 1 nm. The process of double layer formation is commonly modeled by an equivalent circuit of a capacitor in series with a resistor, but depending on the experimental parameters, this model may not be adequate.^109^ A more rigorous mathematical treatment of the capacitive charge buildup in electrolytes is possible but complex.^110^ At low applied voltages, noble metals such as Pt and Au exhibit behavior that is close to the capacitive regime.

#### 2.1.2 Faradaic Regime

In the Faradaic regime, electrodes utilize redox reactions with the electrolyte, i.e. the transfer of electrons to and from species in the electrolyte as the mechanism of transduction.^111,112^ Ionic species are therefore either produced or removed from the electrolyte at the electrode surface, which can lead to the formation of significant concentration gradients in the vicinity of the electrodes. The most commonly observed electrochemical reaction is the electrolysis of water. This typically occurs when the applied voltage exceeds 1.23 V. However, the voltage range that can be applied without water breakdown, is highly dependent on the electrode material.^113,114^ For example, the water windows of platinum and carbon electrodes largely overlap, but the carbon–water window is almost 0.5 V wider than the platinum water window.^115^ Additional reactions in physiological media lead to anodic chlorine gas evolution and cathodic and hydroxide ion evolution. Also, significant oxygen reduction in many commonly used neurostimulation electrode materials has been reported,^116^ leading to hypoxic regions near the electrode surfaces and H_2_O_2_ evolution. The formation of concentration gradients in the electrolyte and unwanted electrochemical byproducts can significantly affect the health and functionality of neural tissue, corrode or degrade the electrodes and therefore needs to be carefully characterized.^108,117–119^ Often a distinction is made for “pseudo-faradaic” electrodes, characterized by electrochemical reactions involving the electrode material itself. A typical electrode in this category is Ag/AgCl^120–122^ (see below).

### 2.2 Operation Considerations

Whether an electrode operates in the capacitive or Faradaic regime depends on factors such as electrode material and morphology, electrolyte composition, applied voltage and stimulation duration, with mixed behavior often observed. The operation mode for a particular electrode design and parameter set can be investigated using electrochemical impedance spectroscopy^108,123^ and cyclic voltammetry.^115^ In most *in vitro* stimulation experiments, the typical arrangement comprises the use two electrodes in a parallel plate capacitor geometry immersed in the same physiological solution and compartment as the cells (see Figure 2).^124,125^ With Pt electrodes, for example, capacitive operation is dominant in high-frequency biphasic stimulation, where the ionic layers at the electrode/electrolyte interfaces are continuously repolarized.^125^ In this scenario the production of undesirable Faradaic reaction side products is minimal. In low-frequency and DC stimulation settings, Faradaic reactions dominate,^125^ leading to the formation of hydrogen peroxide and other electrochemical byproducts, which may affect the tissue.

Several studies^126–129^ have demonstrated potential applications for DC stimulation, however, capacitive electrodes would result in electrolysis of water which is highly undesirable due to corrosive gas formation and pH drift. To address these issues, Faradaic electrodes such as Ag/AgCl are typically used for DC stimulation, but these raise concerns due to the release of Ag. Indirect coupling approaches, where the electrodes are physically separated from the cell medium by a salt bridge, are frequently employed to reduce these effects.^124,130,131^ The setup typically consists of a main compartment containing the biological target immersed in a suitable physiological electrolyte connected to two separate electrochemical half-cells. Each half-cell contains a compartment filled with an electrolyte solution (e.g., saline or a conducting hydrogel) and an electrode (e.g., Ag/AgCl). U-shaped tubes filled with hydrogels connect the electrode half-cells to the main compartment and allow ion flow between the cells. Due to the physical distance between electrodes and biological target, the latter is protected from electrode reaction products allowing safe low frequency and DC stimulation. Finally, it should be mentioned that microelectrode arrays (MEAs) have been extensively used to offer high spetial resolution interfacing with tissues both *in vitro* and *in vivo*.^132–137^

### 2.3 Performance Metrics

Electrical stimulation of cells is typically achieved through the application of repetitive pulses.^37^ Figure 3 shows an example of a monopolar pulse stimulation profile, characterized by amplitude, offset (zero in this example), pulse length, frequency (number of pulses per second), and duration of application. Bipolar waveforms include pulses with the opposite amplitude. Less often, AC waveforms, characterized by amplitude, offset, frequency, and duration of application are used. Similarly, DC stimuli are characterized by amplitude and duration of application.

**Figure 3:**
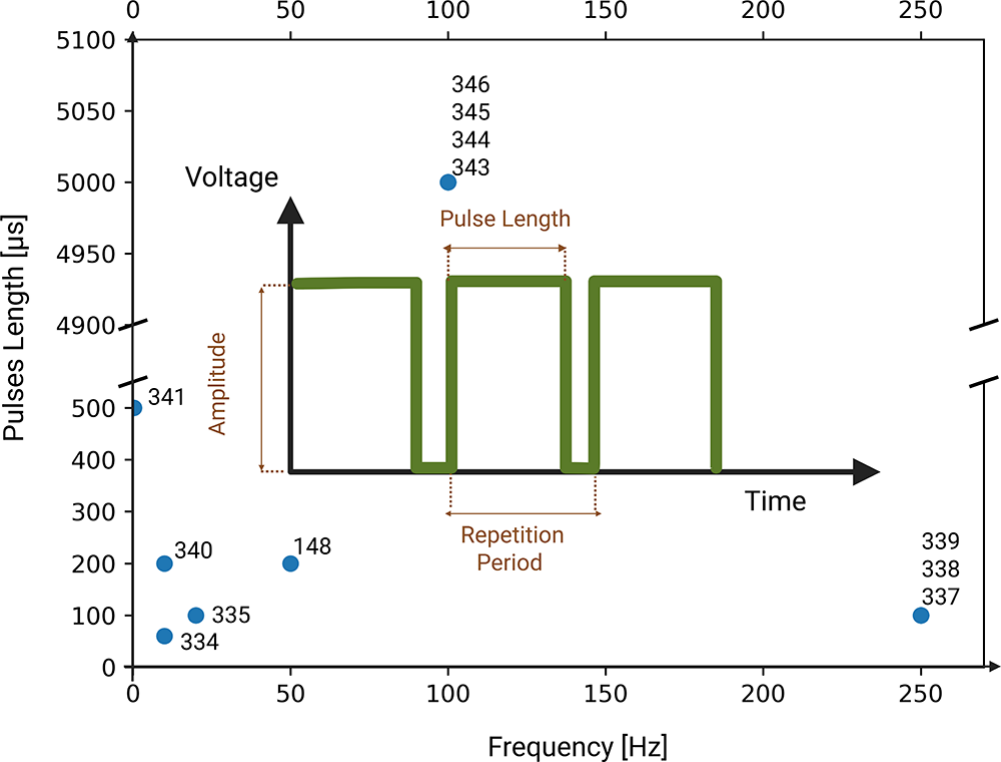
Examples of different stimulation parameters used in literature. The inset shows a typical voltage profile used in monopolar pulsed stimulation. Frequency in this context refers to number of pulses per second (inverse of repetition period), the numbers next to the blue dots refers to the references cited in this review paper. Created in https://BioRender.com.

#### 2.3.1 Electrochemical Impedance

The electrochemical impedance measures the degree of opposition to current flow upon the application of a voltage, and is one of the defining characteristics of stimulation electrodes.^111^ The impedance *Z* is defined by Ohm’s law *V* = *Z I*, where *V* is the complex voltage and is *I* the complex current. Thus, impedance is also a complex number where the magnitude is a resistance (measured in Ohms), and the argument is the phase angle between current and voltage. The electrochemical impedance is frequency dependent and typically electrodes are benchmarked for their impedance at 1 kHz.^138,139^ Impedance generally correlates with the electrode surface area with smaller electrodes producing greater impedance and poorer electron-to-ion transduction.^18^ Mixed ionic-electronic conductors and textured electrodes with high effective surface areas are used to lower impedance.^140^

#### 2.3.2 Charge Storage and Charge Injection Capacity

The charge storage capacity (CSC)^141^ represents the ability of an electrode to store charge during stimulation. It describes the amount of charge that can be delivered and the effectiveness of the stimulation; thus, electrodes with a high charge storage capacity can deliver larger amounts of charge, allowing for higher-level and longer-lasting stimulation. This is particularly important in cases where high charge densities or long stimulation durations are required, such as in deep brain stimulation for the treatment of Parkinson’s disease.

The charge injection capacity (CIC) refers to the ability of an electrode to inject charge without causing irreversible reactions at the electrode-tissue interface. This parameter determines the maximum amount of charge that an electrode can safely inject into a tissue without causing damage or adverse effects. A substantial body of evidence suggests that measurements of CIC are affected by factors such as solution composition, gases present in the solution, and other variables.^142^

#### 2.3.3 Electrode Stability and Foreign Body Response

Stability of the stimulation electrodes is an important consideration, especially when conducting chronic *in vivo* stimulation.^143^ The stability can be compromised by a variety of factors such as fabrication defects, electrochemical reactions, and mechanical stress.^144^ Additionally, biological responses to the presence of a device such as inflammatory reactions after implantation *in vivo*, can further compromise electrode integrity.^145^ After implantation, rapid protein attachment to the electrode surface occurs, followed by the development of a persistent immune and glial response resulting in encapsulation and thus in an increase of the electrochemical impedance, a process which is often referred to as foreign body response.^146^ It has been suggested that electrical stimulation can modulate the foreign body response.^147,148^ Electrodes, should therefore be developed to be resilient to corrosion, delamination, swelling, dissolution, and mechanical strain and the biological response post implantation should be closely monitored.^149–151^

### 2.4 Electrode Materials

Historically, noble metals such as gold (Au), platinum (Pt), and platinum–iridium (Pt–Ir) have been used extensively in the electrical stimulation of biological tissues due to their biocompatibility and their relative resistance to corrosion.^152,153^ Nonetheless, their poor electrochemical properties,^38^ including high impedance (especially at low frequencies) and low charge storage capacity have limited their use. This imposes the need to use high currents during electrical stimulation, which in most cases leads to operating voltages beyond the water window of the electrode material,^149^ thereby leading to corrosion and the subsequent generation of toxic byproducts.^154^ To allow for stable, effective, energy-efficient, and safe electrical stimulation, a trade-off between electrode material and geometry is essential to ensure the necessary electrochemical performance, including low impedance and high charge injection capacity.^38,107^

Hence, engineering the electrode material has been of utmost importance, with strategies that span from surface modification,^155–159^ such as roughening and increased porosity, to the development and investigation of emerging materials, such as oxide coatings and conducting polymers. The latter are based on mixed ionic and electronic conduction and enable low impedance and high charge injection capacity even when utilizing small electrodes. In this section, we outline the various materials used for stable electrical stimulation, including classical and emerging electrode materials.

#### 2.4.1 Sliver/Sliver Chloride (Ag/AgCl)

Ag/AgCl maintains a stable and predictable electrode potential with minimal polarization, even under current flow, allowing for low impedance and high current densities in electrical stimulation.^160^ The electrochemical reactions involved upon electrical stimulation comprise simple and reversible redox reactions (Ag­(*s*) + Cl– → AgCl­(*s*) + e–, AgCl­(*s*) + e– → Ag­(*s*) + Cl−). In physiological electrolytes silver is oxidized under anodic conditions to form insoluble silver chloride and reduced back to elemental silver under cathodic conditions.^107,161^ Given their nonpolarizable characteristics, Ag/AgCl electrodes are widely used as reference electrodes in electrophysiology for the recording of biopotentials.^160,162,163^ Because Ag ions are cytotoxic,^164–166^ Ag/AgCl electrodes are mostly limited to applications outside the body and represent the gold standard in noninvasive clinical wearable or skin applications in different dry or wet (with the use of electrolyte gels) configurations.^167–169^

Examples of using Ag/AgCl electrodes comprise the study of electrotaxis of various cell types, such as macrophages, fibroblasts, and immune cells, in response to electrical fields.,^170–172^ surface electromyography (sEMG),^173,174^ and transcranial electrical stimulation.^175–178^ Although widely used, Ag/AgCl electrodes lack long-term stability due to drying of the electrolyte gel or changes induced by sweat.^163,179^ This motivated the development of new electrodes that use Ag-based composites with TiN,^180^ carbon-based materials,^173,181^ and conducting polymers such as PEDOT:PSS.^182^

#### 2.4.2 Platinum (Pt)

Pt is a noble metal that exhibits capacitive behavior at low voltages. It can exhibit pseudocapacitive behavior due to the formation of oxide layers as well as the adsorption of hydrogen on its surface. Oxide formation occurs at an overpotential of 200 mV vs Ag/AgCl, which is well within the water window of the electrode.^114^ The oxygen for this particular reaction originates from the water in the electrolyte and is independent of the dissolved atmospheric oxygen.^183^

Plain Pt offers poor electrochemical properties for electrical stimulation, with reported CIC values typically ranging from 0.1 to 0.15 mC/cm^2^ under different stimulation conditions.^184,185^ Surface modifications to enhance its electrochemical properties comprise mainly postprocessing steps, such as roughening via redox pulsing in sulfuric acidic solution, including perchloric acid;^186^ the formation of pores or nanostructures by the chemical reduction of Pt using a fixed potential in an aqueous Pt solution in formic acid^187^ or by fully electrochemical-driven processes;^188,189^ or by the electrochemical and anisotropic etching of Pt while DC pulsing in phosphate-buffered saline (PBS) solution.^190^ In the first case, impedance reductions of down to ∼ 1 Ω·cm^2^ at 1 kHz were achieved, and a CIC of up to 1.0 mC/cm^2^,^191^ which demonstrate significant electrochemical improvements in platinum electrodes for normal stimulation application. In the case of Pt nanostructures, the realization of nanostructured morphologies, spanning from small grains and pyramids to nanoflakes were reported, thereby enabling impedances down to 0.72 Ω·cm^2^ at 1k Hz and CICs of up to 4.39 mC/cm^2^ with good stability during long-term stimulation.^192^ With similar performance, laser-roughened Pt microelectrodes demonstrated stability, maintaining performance under biphasic current amplitude of 250 μA.^193^ Additionally, Pt-black coatings have shown to significantly reduce and enhance CIC up to 1.9 mC/cm^2^ compared to unmodified platinum electrodes.^194^ A NanoPt-coated electrodes exhibit stability at a charge density of 1.5 mC·cm^–2^, with only minor cracking observed post stimulation.^188,195^

Pt is often used in its alloy composition with Ir as Pt–Ir, as it improves mechanical strength and electrochemical performance.^196–198^ Depending on the coating technique used, Pt–Ir electrodes can exhibit diverse electrochemical properties. For example, electrodeposited Pt–Ir electrodes have shown higher CSCs compared to uncoated Pt both in benchtop and in vivo measurements by cyclic voltammetry results,^196^ while sputtered Pt–Ir thin films have shown CSCs of up to 22 mC/cm^2^ and up to 29 mC/cm^2^ when activating the Ir electrochemically.^199^ Nonetheless, when compared to other metals, such as iridium oxide (IrOx), Pt–Ir exhibits lower with CSC, with a maximum value of 3.9 mC/cm^2^.^200^ Pt, and especially Pt–Ir has been used in relevant clinical applications such as in cochlear prostheses,^198,201^ and deep brain stimulation implants.^202–204^ A disadvantage of Pt-based electrodes is the dissolution of Pt, which has been reported even when operating the electrodes within the water window.^38,205^ Additionally, the presence of metal particulates in the vicinity of stimulated neural tissue *in vivo* has also been encountered.^205^

#### 2.4.3 Iridium Oxide (IrOx)

IrOx is often used as a coating on noble metals to improve their electrochemical properties.^206,207^ IrOx coatings feature hydrated oxide layers and cluster-like morphology, which enables volumetric coupling of ionic and electronic charges. By doing so, they achieve high values for both CSC and CIC as well as reversible processes during electrical stimulation. The great advantage is that the oxide is porous and depending on the deposition method used and layer thickness, surface morphology can be controlled.^199,208^ Hence, the charge capacity of IrOx can be easily tuned.^209^ IrOx formation can be achieved by different methods, including activated iridium oxide films (AIROFs),^200,210^ sputtered iridium oxide films (SIROFs),^208,211^ and electrodeposited iridium oxide films (EIROF).^212–214^

The operation of IrOx coatings involves the reduction and oxidation between the Ir^3+^ and Ir^4+^ states of the oxide layer.^210,213^ The cathodic CSC increases proportionally with film thickness, ranging from, 78 to 194 mC/cm^2^ for SIROF films with thicknesses between 240 and 770 nm, respectively. The CIC, which reflects the ability of the film to deliver charge during stimulation, generally increases with surface area and film thickness.^208,215^ Depending on the pulse parameters and electrode geometry, CIC values for SIROF have been reported in the range of 1–9 mC/cm^2^.^208,216^ Furthermore, IrOx has been demonstrated to exhibit excellent biocompatibility in both *in vitro* and *in vivo* settings, and it has also demonstrated a high level of resistance to corrosion,^38,208,217^ making it a suitable material for clinically relevant applications, such as visual prostheses.^218–221^

#### 2.4.4 Titanium Nitride (TiN)

TiN is a metallic and chemically stable material. TiN has been reported as a suitable material for long-term neuronal cultures for up to 14 days *in vitro*.^222–224^ Furthermore, TiN has been tested by several groups as an electrode coating for both *in vitro* ^225,226^ and *in vivo* neural stimulation applications,^227,228^ being used not only as the main electrode material but also as a coating for noble metals such as Au.^228–230^ The electrochemical performance of TiN can potentially be improved through morphological surface changes. However, reports show that although porous TiN coatings decrease impedance and increase charge injection capacity *in vitro*, the pores are subject to protein biofouling,^226^ leading to an impedance increase, and therefore poorer *in vivo* performance than smooth TiN coatings.^227^

TiN can be deposited via physical vapor deposition methods, such as direct current (DC) magnetron sputtering,^231,232^ and film porosity can be adjusted according to the chamber pressure and temperature during deposition.^233^ While CICs of up to 23 mC/cm^2^ have been reported for electrodes with a surface area of 80 μm^2^,^229^ other groups have reported lower CICs, in the range of 0.87 mC/cm^2^ for 4000 μm^2^ electrodes^38,225^ and 2.2 – 3.5 mC/cm^2^ for ∼ 4.05 cm^2^ electrodes.^234^ These differences can largely be attributed to variation in film porosity and surface roughness resulting from different deposition techniques. Several reports^225–228,234^ indicate that TiN electrodes generally exhibit lower CICs when compared to coatings such as IrOx or PEDOT:PSS.^38,225^

#### 2.4.5 Conducting Polymers

Conducting polymers (CPs) are developed by the (usually *p*-type) doping of conjugated polymers. Their open structure enables mixed electronic-ionic conductivity,^235^ which leads to low impedance interfaces with electrolytes.^236^ The electrical, optical, mechanical and biological properties of CPs can be tuned by modifying their chemical structure and level of doping, and by forming composites with other materials.^237–239^ While most CPs exhibit a conductivity lower than that of metals, their mixed conduction, good biocompatibility, mechanical flexibility, and stability under aqueous media make them excellent candidates for use as coatings for noble metal electrodes in electrical stimulation.^140,238,240^ This section describes well-known examples of conducting polymers as shown in Figure 4.

**Figure 4:**
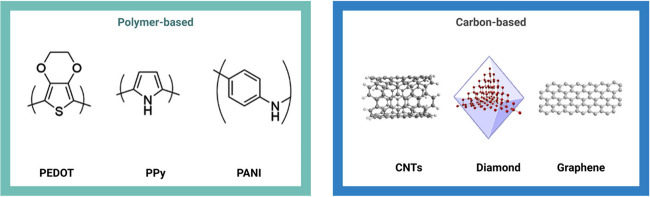
Common polymer-based materials used for electrostimulation: poly­(3,4-ethylenedioxythiophene) (PEDOT), polypyrrole (PPy), and polyaniline (PANI). Carbon-based materials for electrostimulation: carbon nanotubes (CNTs), diamond, and graphene.

##### 2.4.5.1 Poly­(ethylene dioxythiophene) (PEDOT)

PEDOT is polymeric semiconductor that is doped to yield stable conducting polymers with good biocompatibility, optical transparency, high conductivity, and physical and chemical stability.^241^ The most often used dopant is polystyrenesulfonate (PSS). PEDOT:PSS is a organic mixed ionic-electronic conductor (OMIEC) that arises from the presence of two physically distinct, yet closely interpenetrated phases: a PEDOT-rich phase that serves as the hole transport region and a PSS-rich phase that facilitates ion transport.^235^ Given the insulating properties of PSS^–^, the conductivity of PEDOT:PSS drops as low as 1 S/cm^242,243^ in their untreated state. However, various post-treatment methods can significantly enhance its conductivity to values reaching 4000 S/cm.^243,244^ For example, secondary dopants or conductivity enhancement agents (e.g., glycerol, ethylene glycol, or dimethyl sulfoxide - DMSO)^242,245^ are added to the PEDOT:PSS solution before film deposition. Other approaches include acid (e.g., H_2_SO_4_) or solvent (e.g., DMSO) postdeposition treatments.^246^

There are two main methodologies to synthesize PEDOT:PSS, by using chemical oxidative (e.g., oxidizing agents such as iron-based oxidants) or electrochemical polymerization. Chemical oxidative polymerization consists of the polymerization of the PEDOT monomer in the presence of the PSS electrolyte in aqueous media, using an iron-based catalyst and generally in the presence of ammonium persulfate for ensure a proper oxidant environment.^247^ PEDOT:PSS electropolymerization is based on the oxidation and polymerization of the EDOT monomer in the presence of the Na^+^PSS^–^ counterion.^158,241,249^ Both methodologies lead to films that are mixed ionic-electronic conductors and offer low impedance and high charge injection capabilities.^38,239^ Further enhancement of electrochemical properties can be obtained by increasing surface roughness.^250,251^

PEDOT:PSS is receiving a great deal of attention as an electrode for bioelectronics. While CICs of up to ∼ 1.88 – 15 mC/cm^2^ have been demonstrated for PEDOT:PSS electrode coatings on noble metals electrodes,^38,252,253^ electrodes solely composed of PEDOT:PSS have reported CICs around 3.6 mC/cm^2^_,_ with some cases reaching up to 15 mC/cm.^2,38^ Due to its high volumetric capacitance, PEDOT:PSS enables more efficient electric field delivery at low-frequency, outperforming traditional metal electrodes.^125^

One approach to further optimizing electrochemical properties of PEDOT-based CPs involves engineering nanostructured PEDOT layers on electrode surfaces. Surfactant molecules serve as template in this process, facilitating self-assembly and minimizing electrode impedance.^237,254^ Additionally, PEDOT-based nanotubes have shown a substantial reduction in impedance, showing in turn, a much higher CIC, primarily due to an increased surface area and better electrochemical properties.^255^ PEDOT-based CPs have shown enhanced adhesion on porous Pt- or Ir-oxide surface, with no delamination even after 100 days of accelerated aging tests in saline solution.^256^

##### 2.4.5.2 Polypyrrole (PPy)

PPy can also be doped to yield films with a conductivity ranging from 40 to 200 S/cm.^257^ PPy is generally considered insoluble in water, however certain modifications can to enhance its solubility.^258^ PPy was among the pioneering models to clarify the polymerization mechanism of CPs, particularly in understanding oxidation pathways, dopant incorporation, and conductivity evolution.^259^ Besides, PPy coatings on metal electrodes were among the first conducting polymers studied both *in vitro* and *in vivo*. These coatings demonstrated good biocompatibility, and were used in studies of cell adhesion, proliferation and neurite outgrowth.^260–264^ In general, PPy-based polymers, face challenges related to long-term stability, which can limit their application in implantable electrodes.^265^

##### 2.4.5.3 Polyaniline (PANI)

The synthesis of PANI can be achieved by chemical oxidative or electrochemical polymerization in an aqueous media with various of surfactants.^237^ This process facilitates adjustment of the film’s structural characteristics at the nanoscale, enhancing its effective surface area.^237,266^ Different polymerization conditions and therefore oxidation levels, can produce different PANI forms, including the reduced leucoemeraldine base, half-oxidized emeraldine base (PANI-emeraldine base), and fully oxidized pernigraniline base.^267^ PANI based conducting polymers have been utilized in the creation of flexible and cost-effective bioelectrodes for neural sensing and stimulation applications.^268–270^

#### 2.4.6 Other Carbon-Based Materials

Carbon allotropes such as diamond, graphene and carbon nanotubes have attracted significant attention in the field of neuromodulation due to their remarkable electrical conductivity and mechanical strength^271^ (see Figure 4). The incorporation of these materials into composites or coatings can significantly enhance the electrode performance for neural stimulation.^272^

##### 2.4.6.1 Diamond

Diamond is a highly chemically stable and biocompatible material,^273^ but its hardness and lack of ductility pose challenges in device fabrication.^274^ Diamond-based devices, including ultrasmall carbon electrodes, 3D-nanostructured boron-doped diamond (BDD)^275,276^ and hybrid diamond/carbon fiber microelectrodes,^277^ have demonstrated significant potential in neural electrical stimulation. Moreover these diamond based devices have been found to enhance photoelectric stimulation,^278^ improve neural cell attachment and survival, and enable multimodal electrical/chemical neural interfacing. A number of publications have reported high charge injection capacity and good cytocompatibility of nitrogen-doped ultrananocrystalline diamond (N-UNCD) electrodes, which are thus great for neural stimulation and for promoting stem cell growth.^279,280^ Additional studies have shown improved charge injection capacity and stability of platinum and boron-doped diamond (BDD) electrodes with diamond coatings.^275,281^ Flexible, diamond-based microelectrodes have also been developed for neural sensing, demonstrating better sensitivity and stability than traditional electrodes.^282^ Challenges in the fabrication, flexibility, and chronic stability of diamond based electrodes remain.^283^ Still, its exceptional chemical stability and biocompatibility make it a promising material for long-term neural implants.^274^

##### 2.4.6.2 Graphene

Graphene is a two-dimensional single-layer sheet of sp2-hybrid carbon atoms arranged in hexagonal lattice.^284^ This material is widely recognized for its outstanding electrical conductivity, biocompatibility,^237,285^ and adaptability for surface modifications, making it a strong candidate for biomedical applications. In biomedical applications, it maintains stability in biological environments^286–288^ and shows optical transparency that enables the integration of multiple modalities, such as electrophysiological recording and optical imaging, within a single platform.^237,289^ However, its use is limited by their poor mechanical stability and challenging fabrication.^290^ Traditional mechanical exfoliation methods for obtaining graphene are inefficient and time-consuming, while chemical vapor deposition (CVD) provides large-scale, high-quality graphene but is unsuitable for polymer-based substrates.^237,291^ Recent advancement in solution-processable graphene fabrication techniques have emerged, leading to electrodes with lower impedance, improved CIC, and enhanced stability under physiological conditions.^292^ Ongoing research focuses on optimizing graphene’s biocompatibility, functionalization, and integration with other materials to enhance its use in neuroscience and clinical application^293,294^

##### 2.4.6.3 Carbon Nanotubes (CNTs)

CNTs have shown great potential as neural electrodes due to their unique properties, including a high charge capacity and the ability to be chemically modified for enhanced biocompatibility.^286^ Their tubular configuration, charge injection capacity, and potential for chemical modification make them well-suited for neural interfacing. One advantage of CNTs is their ability to undergo surface modification, which enhance their biocompatibility and functional properties, thereby improving performance in neural interface.^237^ Several fabrication techniques enable CNT integration into neural electrodes.^295,296^ Electrochemical deposition allows CNT coating to be applied to traditional tungsten and stainless steel electrode wires under mild conditions, enhancing their impedance and charge transfer properties.^237,297^ Additionally, CVD techniques facilitate the direct formation of CNT structures on the tips of quartz-insulated platinum/tungsten electrodes, ensuring high mechanical stability.^237,298^ Multilayered polypyrrole-coated CNTs significantly enhance electrode performance.^299^ IrOx-CNT hybrids show increased charge capacity and cyclability.^300^ Electrodeposited polypyrrole/CNT composites demonstrate high charge injection limits, low impedance and improved tissue responses *in vivo*.^237,301,302^ CNTs also serve as effective platform for biosensors and drug delivery systems.^303^

In this section we discussed the nature of the signal transduction process and highlighted the mechanism of operation of different electrodes. These are critical considerations for controlling the stimulation delivered to cells. As we will see in the following section (Section 3) , artifacts arising from electrochemical reactions at electrodes can mask the phenomena being investigated.

## 3 Effects of Electrical Stimulation

Electrical stimulation causes some instantaneous effects on cells, for example it changes the membrane potential and leads to the firring of action potentials. However, some biological outcomes of stimulation, such as preferential cell migration toward one of the electrodes, manifest themselves at longer time scales and often after prolonged application of the stimulus. The mechanism of action responsible for these outcomes is not always clear.^304^ We describe here common outcomes of electrical stimulation and discuss mechanisms, with emphasis on *in vitro* studies of neurons.

### 3.1 Examples

#### 3.1.1 Applied Fields Depolarize the Cell Membrane and Induce Action Potentials

The field of electrophysiology deals with the study of the electrical properties of cells and tissues. Neurons, and their ability to fire action potentials, play a central role in electrophysiology.^305–307^ The effects of electrical stimulation of neurons underlie the majority of bioelectronic medicine applications today, including DBS discussed in the introduction.

Neurons are polarized cells, where a so-called resting potential of approximately – 70 mV (inside negative compared to outside) is established across their membranes. This is caused by the action of K^+^ channels and Na^+^/K^+^ pumps,^308–311^ which lead to a higher concentration of Na^+^ and Cl^–^ outside the cell and higher K^+^ concentration inside the cell^312^ (see Figure 5). If the membrane is depolarized to a threshold voltage (approximately −50 mV), usually closed voltage-gated Na+ channels open and this leads to the generation of an action potential,^312^ described below. Conversely, hyperpolarization of the cell membrane decreases the likelihood of an action potential being initiated.^313^ Depolarization and hyperpolarization are graded responses that vary based on the magnitude of the received stimuli, which are usually excitatory or inhibitory neurotransmitters.^305^ Electrical stimulation can also be used to depolarize and hyperpolarize cells. Extracellular cathodic stimulation tends to be excitatory, as negative ions are pushed toward the cell membrane, leading to its depolarization.^314^ The converse is true for anodic stimulation. For details of the polarity, waveforms, and other factors affecting depolarizing cells using electrical stimulation see.^314^

**Figure 5:**
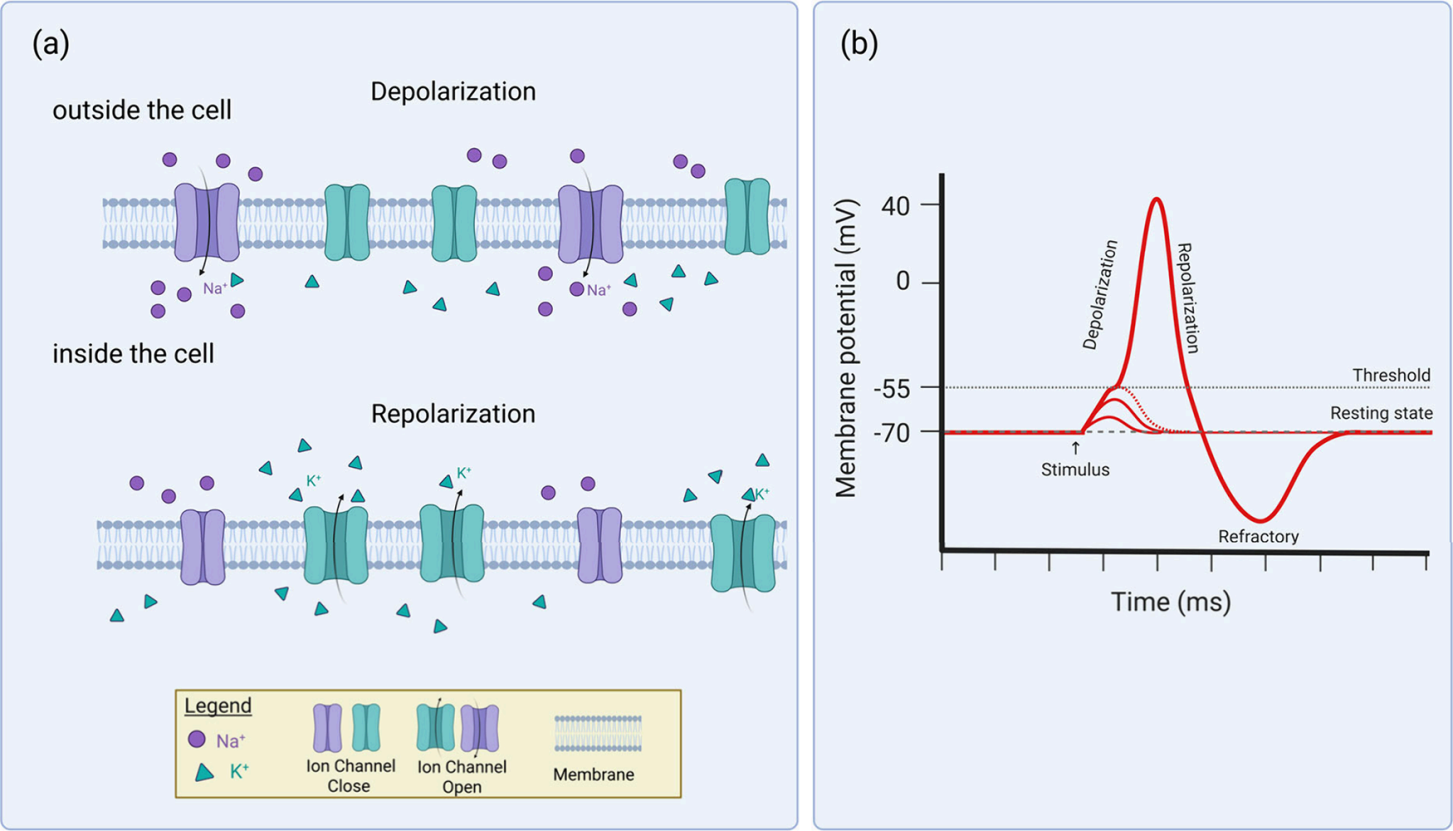
(a) Key stages: Depolarization, Repolarization, (b) Illustration of the action of ion channels during depolarization and repolarization. These processes are central to understanding how cells, particularly excitable cells like neurons and muscle cells, respond to electrical stimulation. Created in https://BioRender.com.

Once the threshold potential is reached and voltage-gated Na^+^ channels open, rapid influx of Na^+^ ions into the cell takes place. As a result, the transmembrane potential can reach approximately +30 to +40 mV.^315^ This reversal of membrane polarization propagates across the axon and results in release of neurotransmitter into the synaptic cleft, and this can lead to excitation or inhibition of a postsynaptic neuron, or muscle contraction. The change in membrane potential triggers the closing of the voltage-gated Na+ channels and the opening of voltage-gated K+ channels, allowing K+ ions to exit the cell and eventually restore the resting potential.^313^

In neural networks, action potentials serve as the fundamental unit of information transmission.^313,316,37^ The action potential is an ″all-or-none″ occurrence, meaning that typically the response is not thought of as “graded” (does not depend on the magnitude of the stimulus).^316,317^ Instead, information is encoded through the frequency of firing action potentials.^312^ When an electric stimulus of sufficient magnitude is applied to a nerve, it can trigger the depolarization of the cell membrane, causing the propagation of an action potential along the nerve fiber.^316,318,319^ However, in nerve bundles, stronger stimuli can modulate the amplitude of the compound action potential as they recruit more neurons with varying threshold potentials.^320,321^ Finally, it is worth mentioning that glial cells are generally thought of as polarized nonexcitable cells, and their resting membrane potential is regulated in a manner similar to that of neurons. However, the excitation events described below typically do not apply to glial cells.^305^

#### 3.1.2 Applied Fields Induce Membrane Permeabilization

Electroporation, which involves the formation of transient pores^57^ in cell membranes due to applied electric fields, is used in gene transfection^322–325^ (see Figure 6). This process, referred to as reversible electroporation, is distinct from irreversible electroporation, which aims to cause cell death.^57^ An alternate term, ″electropermeabilization,″^326^ has been proposed to describe the process of rendering the cell membrane permeable without the formation of pores.^57^

**Figure 6:**
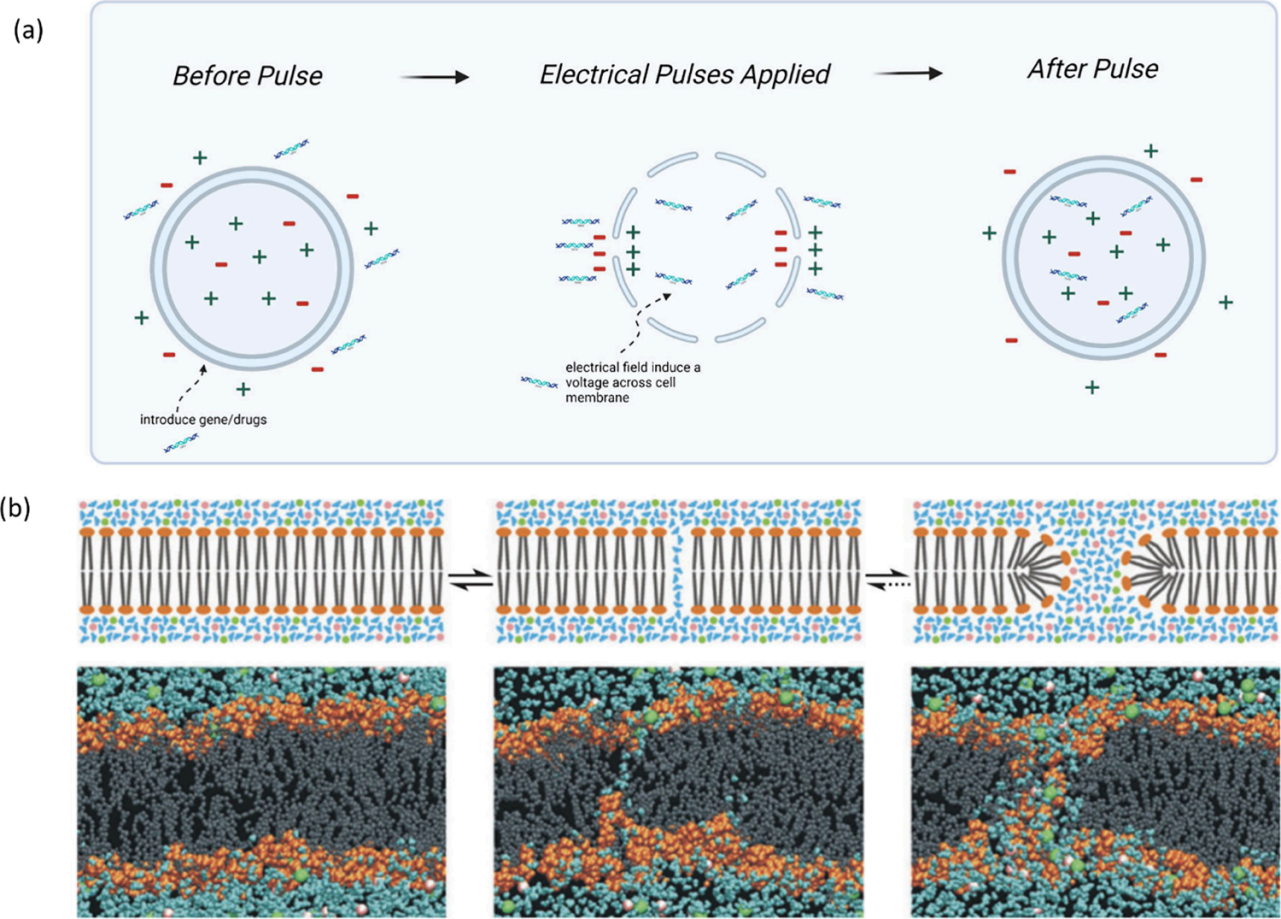
(a) Diagram illustrating the process of electroporation, where an externally applied electric field induces the formation of transient pores in the cell membrane, enabling the transport of molecules across the lipid bilayer. Created in https://BioRender.com. (b) An idealized representation (top) highlights the conceptual steps, while an atomic-level simulation (bottom) reveals molecular interactions and structural changes in the bilayer under an electric field oriented perpendicular to the bilayer. Reproduced with permission from ref 62. Copyright 2014 Annual Reviews.

The kinetics of electroporation-induced transmembrane transport can be divided into five stages:^57^ the initiation of the permeable state, its expansion, stabilization with partial recovery, the resealing of the membrane, and residual memory effects. These processes are highly dependent on the electrical stimulation parameters, such as field strength, pulse duration, and frequency. Electroporation is typically performed in extracellular media with low conductivity.^57^ While most pores reseal within milliseconds to seconds after the cessation of electric stimulation, irreversible electroporation is employed in applications like nonthermal tumor ablation. High electric field strengths typically kill a fraction of the cells, with an efficacy that depends on cell type. This cell death is caused by the inability to restore membrane structure and barrier function, leading to cell membrane rupture, ionic imbalance, and loss of cellular components.

At the molecular scale, electroporation involves electrocompression, where the electric field reduces the thickness of the bilayer membrane and promotes hydrophobic defects, which nucleate into pores due to thermal fluctuations and electric stress. These pores flow as electrostatic forces repel lipid headgroups and are stabilized by water molecules lining the pores interior. Additionally, the electric field disturbs the balance of hydrophilic and hydrophobic interactions in the bilayer, aligning lipid dipoles and facilitating nucleation through charge accumulation at the bilayer/water interface.

#### 3.1.3 Applied Fields Act on Polar Molecules and Can Induce Cell Death

Living cells contain polar molecules that respond to electric fields by reorientation and, in the case of nonuniform fields, dielectrophoretic transport.^12^ These processes lead to downstream cellular changes that include morphological, structural, and synaptic changes.^327^ TTFs are believed to disturb the assembly of microtubules, structures that separate chromosomes into the daughter cells^328^ during the process of mitosis, ultimately preventing cell division^75,329^ (see Figure 7). Furthermore, TTFs lead to a nonuniform electric field at the cleavage furrow of dividing cells in the late stages of mitosis. This leads to dielectrophoretic transport of polar molecules in the furrow, which culminates in apoptosis and inhibits tumor growth.^75^

**Figure 7:**
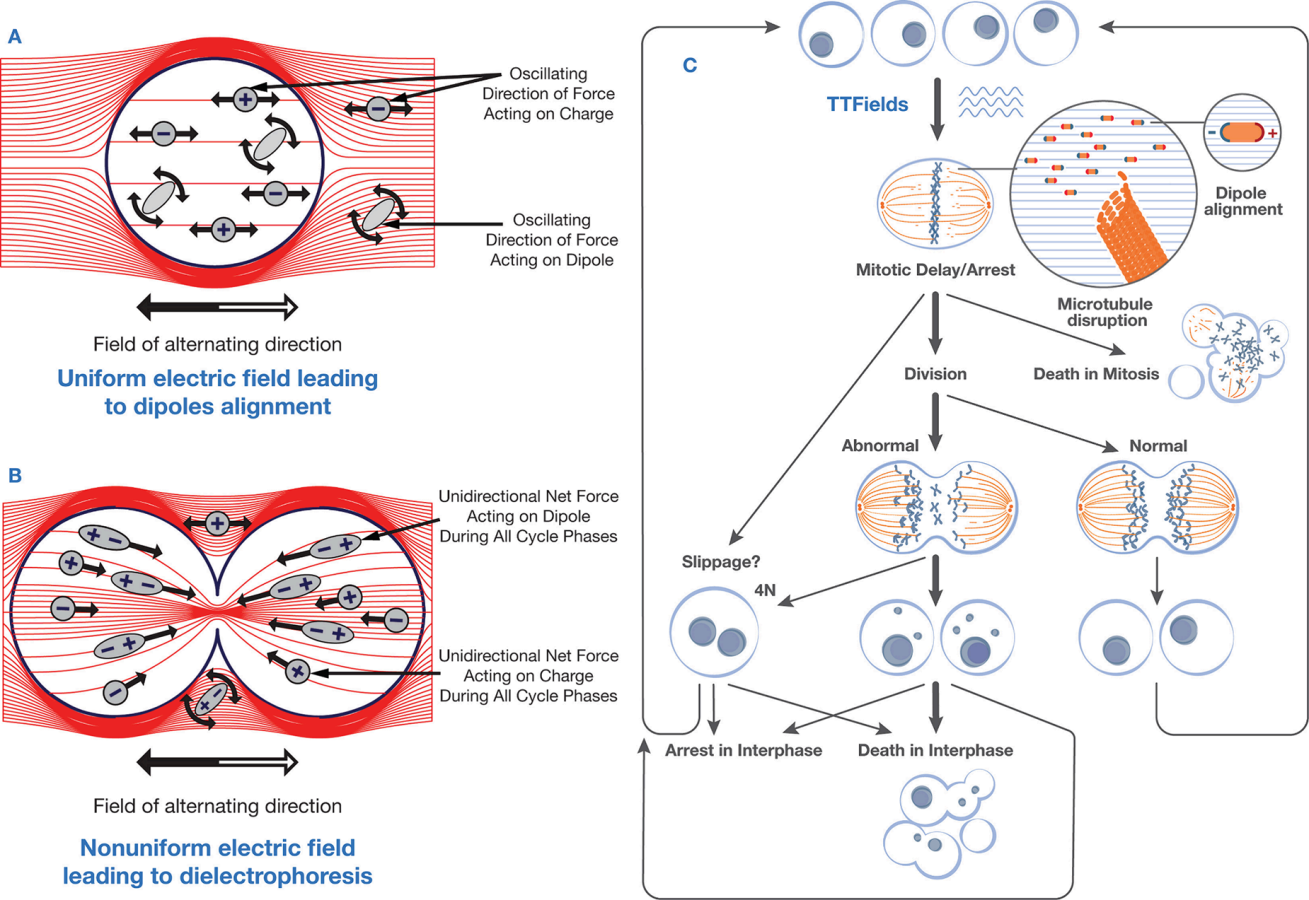
Mechanisms underlying the action of tumor treating fields (TTFs): Molecular polarization disrupts microtubule assembly during mitosis, while dielectrophoresis exerts forces on polar molecules, leading to their redistribution within the cleavage furrow. Reproduced with permission from ref 12. Copyright 2016 Oxford University Press.

Movement and reorientation of charged molecules can affect the local electric field and subsequently alter the neuron’s membrane potential.^330,331^ This process can lead to changes in the neuron firing threshold and modulate the neuron’s response to input signals.^332^ Additionally, altering neurotransmitter-receptor interactions affects the efficacy of synaptic transmission and strength of neuronal communication within the neural network.^37,333^ Thus, affecting molecular polarization by the application of external electric fields contributes to changes in synaptic strength and connectivity.^327,334^

### 3.2 Other Effects

Beyond the examples given above, electrical stimulation of cells has been used to elicit a variety of biological outcomes, discussed below. A snapshot of contemporary research in the field is provided on Table 1 and in Figure 8.

**Table 1:** Examples of Electrical Stimulation Experiments from Contemporary Literature, with Indicative Stimulation Parameters and Effects Observed

Stimulation Method	Indicative stimulation parameters	Pulse length (μs)	Pulse frequency (Hz)	Stimulation duration	Estimated electric field strength (mV/mm)	Cell type	Summary	Electrode material	Adapted from ref.
Pulsed DC	0–200 mV/mm; 60 μs pulses; 10 Hz	60	10	4h/day for 7 days	0–100	NSC	Stimulation shows enhancements in proliferation and differentiation of neural stem cells, which can be related to activity of voltage-gated calcium channels	PEDOT/Cs/Gel scaffolds	^335^
Pulsed DC	200 mV/mm; 100 μs pulses; 20 Hz	100	20	1h, 3h, 8h for 3 or 8 consecutive days	200	NCSC	No stem cell differentiation at 3 days in all cases when cells were cultured on the anode; visible signs of apoptosis when they were stimulated for more than 24 h	Au	^336^
Pulsed DC(Electroporation)	8 pulses; 100 μs duration, 1 Hz; 1300 V/cm	100	1	single sessions	13000	B16–F10 Melanoma cells	Membrane permeabilization; efficient gene delivery	Caliper electrode	^337^
Pulsed AC	Current density of 0.25 mA/cm^2^; 100 μs pulses; 250 Hz	100	250	8h/day for 3 days	—	NSC	Stimulated 3D constructs showed enhanced maturation of neurons	PEDOT:PSS	^338^
Pulsed AC	Current density of 0.1–0.25 mA/cm^2^; 100 μs pulses; 250 Hz	100	250	8h/day for 10 days	—	iPSC	Preferentially assumed a neuronal fate. Interestingly, relatively low glial cell induction was signified by immunophenotyping and RT-qPCR following stimulation	PPy	^339^
Pulsed AC	0–60 mV/mm; 100 μs pulses; 250 Hz	100	250	8h/day for 5 days	0–60	PC12, Schwann	Increased neurite outgrowth from PC12, NGF expression from Schwann via ELISA; 100 mV caused death	PEDOT:PSS	^340^
AC	±5 V; 200 μs pulses; 10 Hz for 0.5s every 2s	200	10	24h/day for 2–3 weeks	—	Mice neurons (DRG), astrocytes, oligodendrocytes	Stimulation applied to astrocytes in coculture increased myelination of axons by oligodendrocytes while LIF – /– astrocytes did not exhibit the effect	Pt	^341^
AC	Charge density of 0.347 mC/cm^2^; 200 μs pulses; 50 Hz	200	50	7h	—	Rat cortex	Comparison of stimulated and unstimulated electrodes, found increased inflammatory signaling under stimulation conditions compared to control, also an increase in plasticity related expression	Pt–Ir	^148^
Pulsed AC	<200 μA with 500 mV, 500 μs cathodic pulses and 125 mV, 2000 μs anodic pulses; 0.29 Hz	500, 2000	0.29	1h	250	NSC	Increased neurosphere numbers in vitro and in vivo (mice) after several days; increase in size of neural stem cell pool; enhanced neurogenesis	Pt	^342^
Intermediate Frequency AC (TTFs)	AC (200 kHz), 1–2 V/cm	Continues	∼200 kHz	∼12h/day	∼1000–2000	NSCLC tumor cells	Phase II study; TTF with pemetrexed improves progression free and overall survival in advanced NSCLC	Insulated electrode	^343^
AC	±800 mV; 100 Hz	5000	100	1h	—	NSC	STC2 upregulation; improves repair mechanism by promoting proliferation of endogenous stem cells	PPy	^344^
AC	800 mV; 5 ms pulses; 100 Hz	5000	100	1h	20–80	NSC	Increased neurotrophic factor expression; increased neuronal differentiation	Graphene	^345^
AC, DC, Pulsed DC	±1 V; 100 Hz	5000	100	24h/day for 4 days; 12h/day for next 8 days	—	NSC	Pulsed DC stimulation best of different methods at neuronal differentiation; AC did not show similar results for ReN cells (measured by staining TUBB3, Nexin, etc.)	PEDOT:PSS	^346^
Pulsed DC	1 V; 100 Hz	5000	100	24h/day for 4 days; 12h/day for next 8 days	100	NSC	PEDOT:PSS was shown to elongate cells under pulsed DC stimulation; potential steering of laminin toward electrode surface	PEDOT:PSS	^347^
DC	250 mV/mm (1–1.5 mA)	Inf	0	3h	250	NSC	Increased neurosphere numbers in vitro and in vivo (mice) after several days; increase in size of neural stem cell pool; enhanced neurogenesis	Ag/AgCl	^342^
DC	50 mV/mm DC (1 mA)	Inf	0	8h	50	Rat neuron	Neurite outgrowth enhanced in conductive SWCNT (regardless of stimulation); even more in combination with electric stimulation	SWCN-composite hydrogels	^348^
DC	250 mV/mm	Inf	0	6h	250	Mice NPC/NPC-derived astrocytes	Cathodal cell migration which was partly inhibited by Ca^2+^ transport inhibition	Ag/AgCl	^129,349^
DC	0, 40, 100, 300 mV/mm	Inf	0	4+h	0–300	Human astrocytes	Anodal migration which increased with field increasing. Elongated morphology and loss of circularity with stimulation. Effects were decreased with PI3K and ERK inhibitors	Ag/AgCl	^350^
DC	0, 4, 40, 400 mV/mm	Inf	0	12+h	0–400	Rat astrocytes	Anodal cell migration reversing direction when polarity of stimulation was reversed. Increased cell proliferation, alignment of axis of cell division	Ag/AgCl	^127^
DC	10, 500 mV/mm	Inf	0	24h	10, 500	Rat astrocytes and DRG neurons	Astrocytes which were seeded first were aligned perpendicular to the applied field. Subsequently cultured DRG neurons followed this alignment without stimulation	Likely Ag/AgCl	^351^

**Figure 8:**
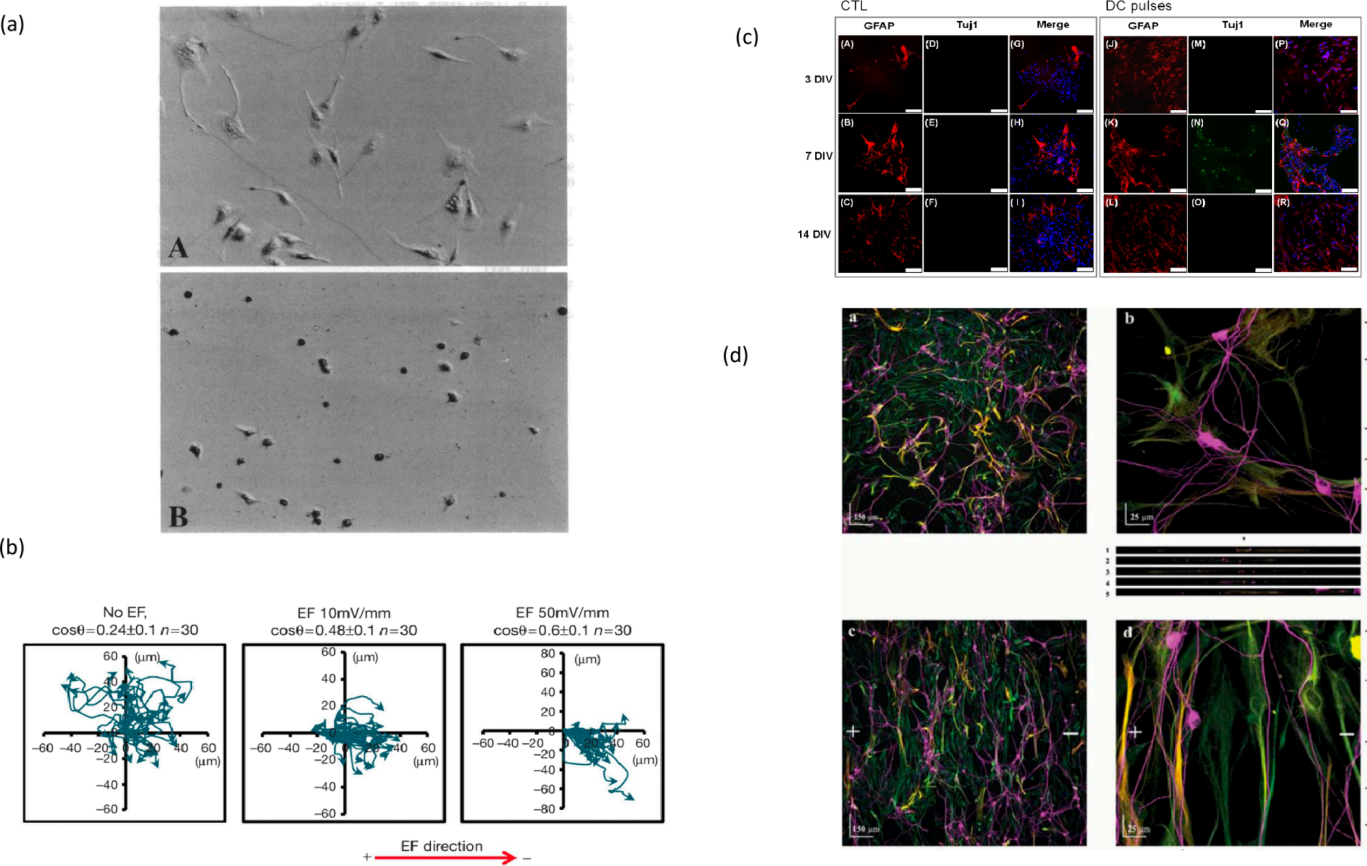
Key cellular responses to electrical stimulation. (a) Endothelial cells show different adhesion when cultured on negatively (A) and positively (B) biased polypyrrole films. Reproduced from ref 352. Copyright 1994 National Academy of Sciences, U.S.A. (b) An applied electric field (EF) directs cell migration toward the cathode. Reproduced with permission from ref 353. Copyright 2013 EMBO Press (Springer Nature). (c) Fluorescence microscopy images of immunostained mouse neural stem and progenitor cells (mNPCs) shows the application of DC pulses inducing differentiation. Cell labeling included GFAP (red), a predominantly astrocytic marker, as well as Tuj1 (blue), a predominantly neural marker. Scale bar: 75 μm. Adapted from ref 354 under the Creative Commons Attribution License (CC BY). Copyright 2016. (d) Alignment of cell structure in response to electrical fields: Neurites (TUJ1+, magenta) align with astrocyte processes (Vimentin+, green; GFAP+, red). Reproduced from with permission from ref 351. Copyright 2006 Cambridge University Press.

#### 3.2.1 Cell Adhesion

Electrical stimulation has been shown to significantly influence cell adhesion.^352^ Initial experiments showed that polypyrrole (PPy) surface charge could regulate cell shape and attachment. The interaction between cells and conducting polymer surfaces is influenced by the redox state of the underlying polymer, as cells can distinguish between oxidized and reduced states. The observation was later confirmed with other cell types and conducting polymer films.^355,356^ One proposed mechanism for this phenomenon involves the change in pH at the film surface during electrochemical reduction/oxidation, which subsequently alters the conformation and adsorption of extracellular matrix proteins like fibronectin.^356^ More recently, the impact of electrical stimulation on a copolymer of PEDOT and poly­(d,l-lactic acid) (PEDOT-*co*-PDLLA) was investigated,^357^ finding that electrical stimulation enhanced fibronectin adsorption and improved fibroblast adhesion and proliferation. Similar results were later seen in PPy films, where positively charged fibronectin adhesion was improved by negatively charging the surface.^358^

#### 3.2.2 Directed Cell Migration (Galvanotaxis)

Galvanotaxis is a commonly reported effect, where cells migrate preferentially toward the anode or cathode in an electric field. The effect has been observed in many cell types. Although not uncommon, galvanotaxis is more often reported in precursor/stem cells rather than fully differentiated cell types, which may be linked to its potential role in guiding cell migration in a developing embryo. Naturally occurring electric fields of approximately 3 mV/mm have been shown to guide neuroblast^353^ or neural crest cell^359^ migration. Reports also involve neural precursor cells (NPCs).^129,360^ Moreover, several studies have demonstrated the movement of injected neuronal cells from the original source owing to an applied electric field.^361–363^ Similar results are seen in astrocytes in a ‘dose dependent’ manner.^127^ In addition, during wound healing, electric fields play an important role in guiding cellular migration,^364^ highlighting the significance of electric fields in tissue repair. Migration and directional behaviors are generally characterized by experimental setups which apply DC or low frequency stimulation with long pulses (10 ms at minimum, but commonly longer than 100 ms). Much of the speculation on the mechanisms suggests electrophoretic redistribution of charged cell membrane proteins or growth factors to specific areas of the cell.^365–367^ Other mechanisms may be at play, however. For instance, the CLASP2 pathway, which affects microtubule polarization, is necessary for directional migration of neural progenitor cells.^368^

#### 3.2.3 Cell Differentiation

Endogenous electric fields guide cellular differentiation and maturation^127,369^ with potential applications in stem cell therapies. Given the challenge of stem cell heterogeneity,^370^ use of biophysical cues such as electrical stimulation may provide a more robust method of influencing cell fate than current uses of biological cues such as addition of growth factors. The typical experimental setup for promoting differentiation into neurons involves short pulses (of the order of 100 μs) at frequencies of 100–250 Hz.^371,372^ Another method involves current pulses of similar duration, but at lower frequencies, typically ranging from 10 to 100 Hz. Continuous stimulation over periods ranging from 1 h to 1 week has led to a consistently preferred differentiation of neural stem cells into neurons and oligodendrocytes, with a decreased inclination toward glial fates.^371–376^ Studies have demonstrated that the polarity of electrical stimulation can significantly influence biological outcomes. For instance, culturing neural crest stem cells in direct contact with either the cathode or the anode, and with a second electrode suspended in the media above,^377^ resulted in preferential neuronal differentiation on the cathode, whereas no significant changes in stem cell differentiation were observed on the anode. Electrical stimulation can potentially activate membrane receptors, which further regulate downstream gene expression. A potential mechanism seems to involve an electric field led influx of calcium ions, which was weakened when blocking calcium voltage channels.^376^ Thus, calcium channels may be the link between electric fields and endogenous intracellular pathways which signal neural stem cells to differentiate into neurons.

#### 3.2.4 Directed Neurite/Process Outgrowth

Several papers discuss *in vitro* experiments showing neurite extension in electric fields,^378–380^ with an excellent historical summary in.^128^ Neurites tend to grow toward the cathode or the anode, seemingly depending on cell type, with an increase in neurite sprouting and branching often reported upon stimulation. Many investigations suggested the use of DC electrical stimulation or pulsed DC to treat spinal cord injuries by promoting neurite growth through the injury. To date, mostly DC electrical stimulation has been used on astrocytes and microglia, with effects similar to those observed in neurons. In particular, process alignment was demonstrated when DC electric fields were passed through astrocytic culture.^127,128,351^ In one study, astrocyte process alignment was used to subsequently pattern neurons in the absence of electrical stimulation.^351^ In an *in vitro* study of microglia exposed to the pro-inflammatory compound lipopolysaccharide (LPS) and stimulated with a DC field, changes in protrusion length were observed.^128^

The most frequently evoked mechanism for this effect involves changes in intracellular Ca2+ concentration, and multiple studies show that inhibiting Ca2+ transport or altering Ca2+ concentrations abolishes the effect.^381–383^ Some reports link the effect to microtubule dynamics and Rho-family kinases.^384^ However, to date the specifics of the mechanism remain unknown. More details on putative mechanisms can be found in section 3.3 ‘Putative mechanisms’.

#### 3.2.5 Pro- and Anti-inflammatory Effects

A promising new area of research involves the investigation of the pro- and anti-inflammatory effects of electrical stimulation. Pathological astrocytes can undergo changes and become reactive, commonly characterized by glial fibrillary acidic protein (GFAP) expression.^385^ Microglia, which are the resident immune cells of the CNS, tend to activate and populate the injury site. Various effects have been observed under electrical stimulation. A significant amount of evidence originates from *in vivo* studies, reviewed in,^386,387^ showing stimulation-induced changes in tissue damage, cell density, and the release of inflammatory mediators in both astrocytes and microglia.^146,388–393^ However, other studies did not observe any differences between stimulation and control conditions.^394,395^ It is likely that the differences in the outcomes stem from the variety of stimulation parameters and electrode materials used.

An *in vivo* study employing AC stimulation in a stroke model found that electrical stimulation improved functional recovery and decreased infarct volume with decreased GFAP and microglial staining.^396^ This effect was abolished by introduction of the phosphoinositide-3-kinase (PI3K) pathway. Simultaneously, higher levels of BDNF, GDNF, and VEGF signaling factors were observed under stimulation. GFAP levels in astrocytes were found to change with different stimulation parameters.^127,128^ A mixed culture model was shown to respond to both DC and low-frequency AC stimulation with changes in NMDA receptor subunits, BDNF, and RAB3A expression changes. These collectively promoted network recovery in response to excitotoxic injury,^397^ and glial precursors in culture showed differential gene expression depending on stimulation parameters.^398^

In microglia, in addition to decreased microglial staining, lower levels of pro-inflammatory signaling molecules were observed under stimulation.^399^ Contrary, in another study, no significant changes in inflammatory cytokine levels were observed after DC stimulation. However, there was a significant difference in cyclooxygenase-2 activity, suggesting an inflammatory component of microglial response to stimulation.^128^ Recent evidence from spatial transcriptomics shows differential expression of IL-1B, Neurexin, GFAP, or BDNF in response to stimulation.^148^ Together with the aforementioned mechanistic studies involving pathway inhibitors, use of the novel techniques represents an important step in the field.

### 3.3 Putative Mechanisms

Understanding the mechanisms behind these effects is still an active area of research.^380,400^ We review here some of the most frequently evoked mechanisms that underpin the molecular and cellular responses, including protein transport, calcium signaling, cytoskeletal reorganization, and transcriptional changes, offering insights into the multifaceted effects of electrical stimulation. Figure 9 summarizes these effects at the cellular level, including growth, migration, differentiation and cytoskeletal reorganization.

**Figure 9:**
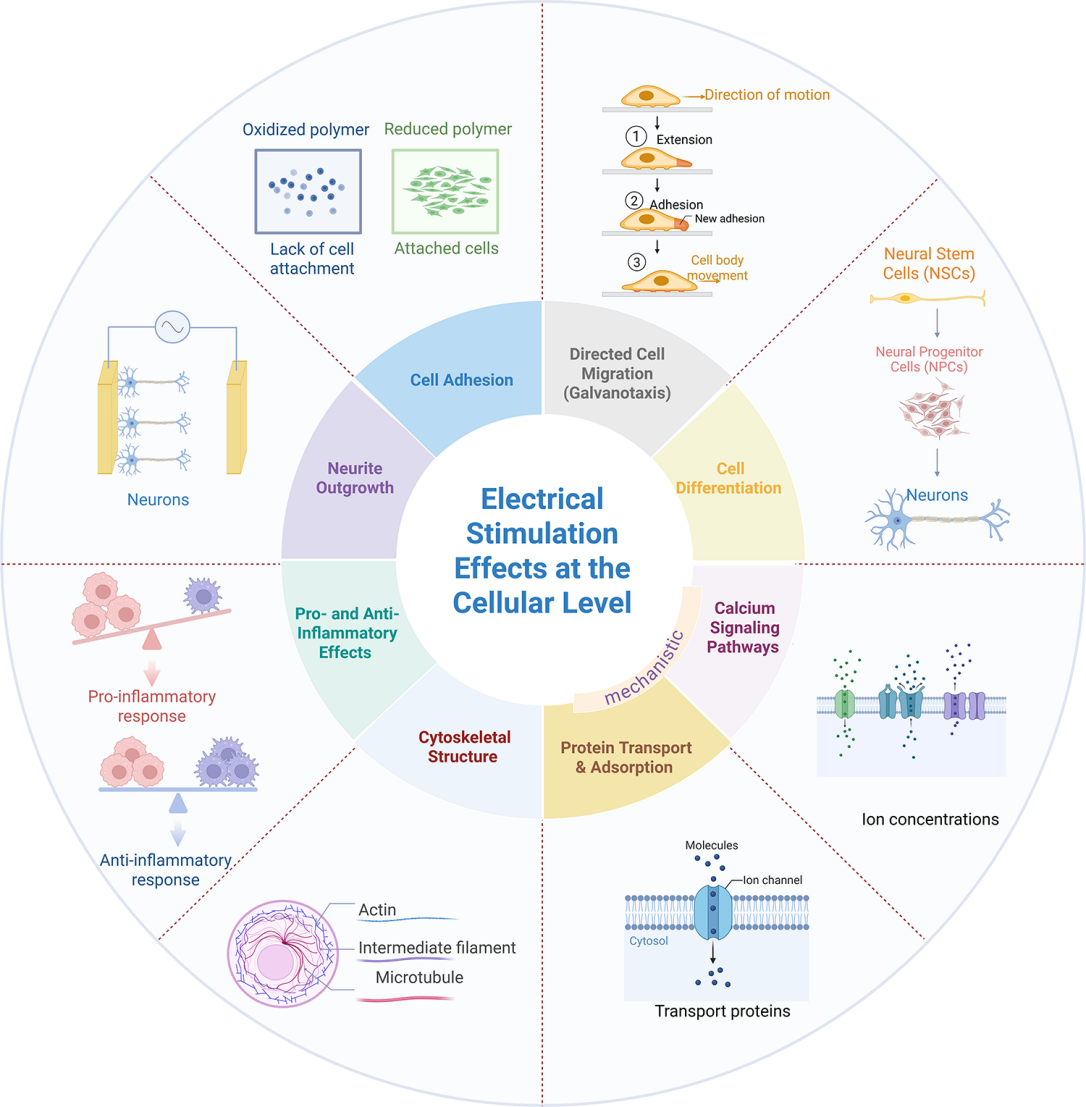
Schematic representation of electrical stimulation at the cellular level. Key effects include cell adhesion, directed cell migration (galvanotaxis), differentiation, neurite outgrowth, pro- and anti-inflammatory responses, protein transport and adsorption, calcium signaling pathways, and cytoskeletal structure reorganization. Created in https://BioRender.com.

#### 3.3.1 Electrophoretic Protein Transport or Protein Adsorption

Electrophoretic redistribution of charged cell membrane proteins or growth factors to specific areas of the cell is believed to be involved in a variety of cellular responses.^365–367^ Changes in the distribution of charged molecules can affect other pathways that lead to differential changes in the membrane, and thus, migratory or directional behaviors. Some studies have suggested that electrostimulation gradients can directly influence the behavior of charged proteins;^401,402^ particularly the conformation of extracellular matrix proteins. This impacts key processes such as cell migration^403^ and cell adhesion in development biology or tissue regeneration.^404^ This is also consistent with the observed directional transport of proteins in electrophoretic systems, which aligns with findings in tissue patterning under electric fields. The phenomenon of cell migration induced by electric fields is unlikely to be solely attributed to the movement of charged particles, as cells can exhibit directional behaviors distinct from those of charged molecules.^405^ For instance, an applied electric field can cause neurites to grow in a particular direction depending on the cell type: toward the cathode in dopaminergic progenitor neurons,^368^ toward the anode in neural crest cells,^406^ or aligning perpendicular to the electric field in astrocytes.^351^ Different signaling pathways in particular cell types may therefore affect behavioral responses to electric fields.

#### 3.3.2 Calcium Signaling Pathways As Transducers of Electrical Stimulation

Calcium signaling pathways have been identified as transducers of electrical stimulation effects, offering a deeper understanding of the cellular responses to external electrical signals. Calcium signaling mechanisms involve calmodulin activation, calcineurin activity and downstream effects on gene expression and protein phosphorylation.^407,408^ The mechanisms suggest that externally applied electric fields can fine-tune calcium fluxes, creating distinct signaling regions. Several studies have noted an increase in intracellular calcium^409^ after electrical stimulation, sometimes in a specific region of the cell,^383^ implicating calcium signaling pathways as a mechanism that transduces electrical stimulation into biological effects in cells; however, the exact causal element remains unclear.

#### 3.3.3 Influence of Electric Fields on Cytoskeletal Structure

A range of studies have demonstrated the influence of electrical stimulation to asymmetrically polarize the cytoskeletal structure,^410^ and found that electrical stimulation caused reorganization of the cytoskeleton in human mesenchymal stem cells, and similarly in rat neurons.^411^ A set of studies from the 1990s indicated changes in the distribution of charged surface receptors of 1–10 Hz, but not higher than 60 Hz AC electrical stimulation.^412,413^ Electric fields have been shown to affect actin reorganization. The application of DC fields to vascular endothelial cells selectively enriches actin formation closer to the cathode,^414^ whereas low-frequency (1–10 Hz) AC fields have been shown to change microfilament organization in hepatoma cells from aligned cables to discontinuous patches, which was not observed at higher frequencies (20–120 Hz).^413^ Increased neuronal cell elongation, measured by the aspect ratio of the cell body, has been demonstrated using pulsed DC 10 ms pulses.^375^ Some studies have proposed models for electrical stimulation mechanisms and the role of the neural cytoskeleton in learning and memory. These studies collectively suggest that electrical stimulation can induce changes in cytoskeletal structure, potentially influencing cellular behavior and function.^415,416^

#### 3.3.4 Transcriptional Changes

Activation of voltage-gated signaling pathways can take place upon electrical stimulation. This activation may lead to increased transcription of proteins that enhance neuronal growth, including ciliary neurotropic factor (CTNF),^417^ vascular endothelial growth factor (VEGF),^418^ stanniocalcin-2 (STC2),^419^ nerve growth factor (NGF),^420^ and brain-derived neurotropic factor (BDNF).^409,421^ In these studies, changes in neuronal growth are generally evaluated several days after stimulation, which is the time scale when transcriptional changes are observed. However, the precise mechanism underlying this upregulation of protein expression is not fully understood.

Emerging evidence suggests that electrical stimulation enhances intercellular calcium levels through the activation of voltage-gated calcium channels and transient receptor potential (TRP) channels. The resulting calcium influx acts as a second messenger, activating downstream pathways such as the calmodulin-dependent protein kinase (CAMK) pathways, which regulate the activity of transcription factors like cAMP response element-binding protein (CREB).^422^ These transcription factors play a critical role in initiating the expression of neurotrophic factors, including VEGF and BDNF.^423,424^

In one study, the effect of electrical stimulation on neuronal differentiation was reduced in the presence of calcium voltage-gated ion channel blockers.^376^ However, neural growth factors such as VEGF^425^ and BDNF^426^ have also been shown to elevate intracellular calcium, indicating a potential feedback loop between calcium signaling and transcriptional activation; it is unclear which of these elements is causal, but it seems to be a combination of the upregulation of neuronal factors, activation of voltage-gated signaling pathways, and changes in ion concentrations that lead to the observed enhanced neuronal growth and differentiation due to electrical stimulation.

### 3.4 A Word of Caution

Some electrodes elicit redox reactions at their surface and can therefore generate drastic pH changes or toxic electrode breakdown products. In most of the studies reviewed here, electrode behavior is not well characterized. In many cases, this issue is avoided by implementing salt bridges between the cell culture and electrodes, which shield cells from toxic compounds. However, there is some evidence that can serve as caution. One *in vivo* study of DC stimulation in the cortex found profound increases in astrocytic GFAP and microglial activation in the vicinity of stimulated electrodes compared to the control,^427^ likely underscoring the dangers of a lack of charge balance and electrochemical reactions at electrodes. To further highlight the likely importance of electrochemical reactions,^428^ it has been reported that directed cell migration in glioma cells was abolished by N-acetyl-l-cysteine and by overexpressing superoxide dismutase in the mitochondria, both of which may be protective from the electrochemical reaction products at the electrode/tissue interface. In addition, some of the observed effects may be electrode-specific, especially when using conducting polymer electrodes. This was highlighted nicely in reference,^352^ where changes in adhesion where observed with conducting polymer electrodes, but disappeared when indium tin oxide electrodes were used. This underscores the complexity of cellular responses to electrical stimuli and emphasizes the importance of standardizing experiments to discern direct effects on cells.

## 4 Summary and Future Directions

In summary, electrical stimulation is already used in many applications of bioelectronic medicine for the treatment of conditions as diverse as heart arrhythmias, sensory loss, and movement disorders. Knowledge in the field advances within communities that focus on neuromodulation, tumor-treating fields, and electroporation. Cells respond to stimulation acutely, through mechanisms involving changes in membrane potential, membrane permeability and orientation/migration of polarized molecules. They show a large diversity of long-term responses, including changes in adhesion, migration, differentiation, process outgrowth and inflammatory effects. These results point to a future where we transition from today’s Bioelectronic Medicine version 1.0, where we use electricity to interface with the nervous system “as is”, to tomorrow’s Bioelectronic Medicine version 2.0, where electrical stimulation is used to locally change the structure and function of the nervous system, then control it.

Several putative mechanisms for these responses have been proposed, however, our understanding is still at its infancy. Significant progress has been obtained through *in vitro* experiments in tissue cultures. Even though these experiments deal with an environment that is greatly simplified compared to the *in vivo* one, there is still considerable complexity to be dealt with. As mentioned in section 3.4, electrochemical reactions and other electrode-specific effects can alter cell behavior, making it difficult to decouple the effects of electrical from chemical stimulation. These effects become pronounced at low frequencies and high applied voltages, as the electrodes can charge up, leading to a collapse of the applied voltage to the electrode/media interface.^125^ Electrodes with very large capacitance were shown to remedy this issue.^125^ Standardizing experimental design, considering the impact of the nature and geometry of electrodes, choosing the appropriate stimulation protocol, and reporting complete information including both voltage and current values during stimulation can help compare data across laboratories and speed up the pace of progress. Measuring the field distribution inside the electrolyte can offer significant insights on the quality of the experimental setup.^125^

Beyond challenges with the experimental setup, key questions remain about the mechanisms involved in determining cellular response to electrical stimulation. For instance, the role of ion channels in mediating complex cellular responses is not fully understood. This is particularly true for their contributions to calcium dynamics, cytoskeletal reorganization, and transcription activation, all of which are critical for cellular responses like proliferation, differentiation, and migration. The key to answering such questions lies in a systematic approach, where cells are stimulated with different amplitudes and frequencies to establish the parameter space within which significant changes occur, and the cellular response is characterized with an arsenal of techniques that include electrophysiology, immunohistochemistry and molecular profiling. Integrating knowledge from the communities of electrophysiology, electroporation and tumor-treating fields is important in this endeavor, as it helps consider different mechanisms that may be at work at the same time.

Most of the experimental work was carried out on neurons, as these cells are directly involved in current neuromodulation approaches for bioelectronic medicine. Increasing evidence, however, indicates the importance of non-neural cells in the physiology and pathology of the nervous system. These cell types include astrocytes, microglia, oligodendrocyte precursor cells (OPCs)/oligodendrocytes, and neural progenitor cells (NPCs). Evidence regarding the effect of electrical stimulation on these cells is sparse, and more research is needed to understand the effect of specific stimulation parameters on their behavior.^386,387^ Astrocytes, one of the main glial cells in the brain, are supportive cells that regulate the neuronal environment by contributing to ion homeostasis, buffering neurotransmitters, energy metabolism, and participating in tripartite synapses.^385,388,429,430^ Although nonexcitable, they have been shown to form networks that can transmit information via calcium ion transients.^431^ Finally, a wealth of evidence points to their roles in nervous system pathology.^385^ Microglia are the resident immune cells of the brain that are capable of destroying pathogens, degrading debris, and clearing damage to the extracellular matrix.^432,433^ Oligodendrocyte precursor cells (OPCs) are cells which canonically give rise to mature oligodendrocytes, which in turn myelinate neurons.^434^ Looking at the impact of electrical stimulation on these cells and their cocultures with neurons will yield valuable information.

Translating knowledge from 2D cell cultures to *in vivo* models poses significant challenges, as the environment is more complex and the electrode configuration becomes more challenging to implement. 3D cell cultures^435^ and organoids^436^ provide intermediate levels of complexity and can help navigate some of the challenges posed in a gradual fashion. Miniaturization can help create smaller yet more sophisticated electrical stimulation systems suitable for *in vivo* work.^437^ Simulation of the generated current/field distribution using finite element models that consider anatomical details^438^ can help quantify the volume of tissue that is stimulated and help understand the results. Finally, the use of responsive materials and soft robotic actuators offer unique opportunities to make implantable electrodes that move inside the body and establish large area and spatiotemporally controlled interfaces in a minimally invasive fashion.^439^

In the future, electrical stimulation will be applied together with other treatment modalities to achieve more effective therapeutic outcomes,^440^ and this will undoubtedly drive developments in the field. This combination approach reflects the growing interest among researchers and clinical practitioners in enhancing treatment results for neurological disorders.^437^ For example, in glioblastoma, combination therapies address existing treatment limitations.^441^ Furthermore, research on conditions such as multiple sclerosis,^442^ stroke,^443^ and substance addiction^444^ suggests that combining stimulation modalities can lead to more effective and targeted therapeutic outcomes. Multimodal treatment strategies, combining electrical, optical, chemical, acoustic, and magnetic stimulation, can facilitate less invasive procedures, improve electrode/tissue interfaces, increase brain accessibility, and achieve higher spatiotemporal resolution.^440,445^

Advanced imaging techniques and the growing body of brain connectivity data will be used to personalize electrical stimulation-based treatments.^446,447^ Large scale imaging data sets have already been shown to link neuronal connectivity with functional outcomes in Parkinson’s Disease.^448,449^ Integrating artificial intelligence (AI) and predictive technologies could enable real-time therapy adjustments, leading to more adaptive treatments.^450^ For example, AI models have been applied to optimize deep brain stimulation settings for epilepsy, significantly improving patient outcomes.^451^ These advancements not only personalize treatments but also reduce the time required for clinical parameter adjustment. Finally, the cross-fertilization of bioelectronic medicine and regenerative medicine is yielding implants that codeliver electrodes with transplanted cells^452^ The transplanted cells in these implants repair damaged or dysfunctional tissue, while the electrodes monitor the process and then interface with tissue that has been repaired. Using the electrodes to control the transplanted cells and speed up the repair process seems to be imminently achievable.
